# *SF3B1-*mutant models of RNA mis-splicing uncover *UBA1* as a therapeutic target in myelodysplastic neoplasms

**DOI:** 10.1038/s41375-025-02740-1

**Published:** 2025-08-26

**Authors:** Jonas Thier, Sophia Hofmann, Katharina M. Kirchhof, Gabriele Todisco, Teresa Mortera-Blanco, Ingrid Lilienthal, Dimitris C. Kanellis, Indira Barbosa, Ann-Charlotte Björklund, André G. Deslauriers, Jiri Bartek, Nikolas Herold, Elli Papaemmanuil, Eirini P. Papapetrou, Eva Hellström-Lindberg, Pedro L. Moura, Vanessa Lundin

**Affiliations:** 1 Center for Hematology and Regenerative Medicine, Department of Medicine Huddinge, Karolinska Institutet, Huddinge, Sweden; 2 Department of Biomedical Sciences, Humanitas University, Milan, Italy; 3 Division of Pediatric Oncology and Surgery, Department of Women’s and Children’s Health, Karolinska Institutet, Stockholm, Sweden; 4 Department of Medical Biochemistry and Biophysics, Science for Life Laboratory, Division of Genome Biology, Karolinska Institutet, Stockholm, Sweden; 5 Global Drug Discovery, Novo Nordisk A/S, Måløv, Denmark; 6 Danish Cancer Institute, Danish Cancer Society, Copenhagen, Denmark; 7 Pediatric Oncology, Astrid Lindgren’s Children’s Hospital, Karolinska University Hospital, Stockholm, Sweden; 8 Computational Oncology Service, Department of Epidemiology and Biostatistics, Memorial Sloan Kettering Cancer Center, New York, NY USA; 9 Department of Oncological Sciences, Icahn School of Medicine at Mount Sinai, New York, NY USA; 10 Tisch Cancer Institute, Icahn School of Medicine at Mount Sinai, New York, NY USA; 11 Center for Advancement of Blood Cancer Therapies, Icahn School of Medicine at Mount Sinai, New York, NY USA; 12 Black Family Stem Cell Institute, Icahn School of Medicine at Mount Sinai, New York, NY USA; 13 Department of Medicine, Icahn School of Medicine at Mount Sinai, New York, NY USA; 14 Department of Medicine, Division of Hematology, Karolinska University Hospital, Huddinge, Sweden

**Keywords:** Myelodysplastic syndrome, Haematopoietic stem cells

## Abstract

Myelodysplastic syndromes with somatic mutations in the splicing factor *SF3B1* gene (MDS-*SF3B1*) result in RNA mis-splicing, erythroid dysplasia and ultimately refractory anemia. Precision medicine approaches for MDS-*SF3B1* remain challenging due to both the complexity of the mis-splicing landscape and its evaluation in disease-accurate models. To uncover novel RNA mis-splicing events, isogenic *SF3B1*^K700E^ and *SF3B1*^WT^ iPSC lines from an MDS-*SF3B1* patient were differentiated into hematopoietic cells and analyzed via unsupervised splicing event profiling using full-length RNA sequencing. This identified *SF3B1*^K700E^-specific mis-splicing of ubiquitin-like modifier activating enzyme 1 (*UBA1*), which encodes a key E1 protein at the apex of the ubiquitination cascade. *UBA1* mis-splicing (*UBA1*^ms^) introduced protein instability and decreased total UBA1 levels, rendering mutated cells susceptible to the small-molecule UBA1 inhibitor TAK-243. Analysis of CD34^+^ RNA sequencing data from an MDS patient cohort confirmed unique and ubiquitous *UBA1*^ms^ in MDS-*SF3B1* patients, absent in other splicing factor-mutated MDS cases or healthy controls. TAK-243 selectively targeted MDS-*SF3B1* primary CD34^+^ cells and reduced mutant cell numbers in colony-forming assays. In contrast, normal hematopoietic progenitor cells were unaffected. Altogether, we here define *UBA1*^ms^ as a novel therapeutic vulnerability in *SF3B1*-mutant cells, introducing UBA1 inhibition as a potential avenue for future MDS-*SF3B1* treatments.

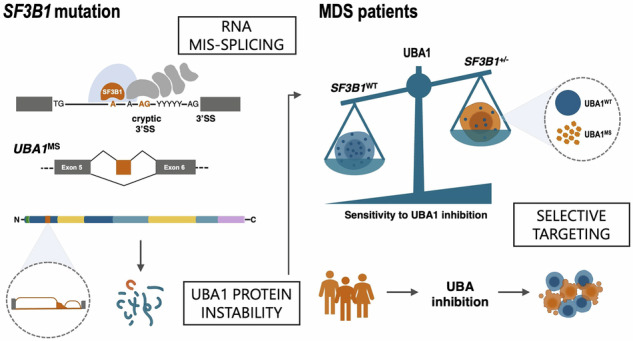

## Introduction

Myelodysplastic neoplasms (MDS) are clonal myeloid malignancies that originate in hematopoietic stem cells, characterized by clonal expansion and ineffective hematopoiesis [1, 2]. Heterozygous, recurrent point mutations in splicing factor genes, such as *SF3B1*, *SRSF2*, and *U2AF1*, are the most common variants in MDS patients and result in widespread splicing defects [3, 4].

MDS with *SF3B1* mutation (MDS-*SF3B1*) constitute a distinct subgroup of MDS, characterized by bone marrow accumulation of mutant dysplastic erythroblasts (ring sideroblasts), erythroid cytopenia, and refractory anemia [5–10]. *SF3B1* encodes subunit 1 of the core RNA splicing factor 3b, involved in processing precursor mRNA to mature transcripts [11, 12]. Missense mutations in this gene alter the interaction of SF3B1 with the pre-mRNA sequence, resulting in extensive cryptic 3' splice site usage and mis-splicing of genes important for hematopoietic and erythroid differentiation, including *ABCB7*, *ALAS2*, *MAP3K7*, *PPOX*, and *TMEM14C* [13–19].

Disease-modifying therapies for MDS-*SF3B1* are limited. Therapeutic target studies using experimental disease models are complicated by the dynamic nature of RNA mis-splicing in MDS: mouse models of *SF3B1* mutation develop anemia but feature distinct RNA splicing from humans [20–22]; primary patient material is heterogeneous, has limited availability and in vitro longevity; and cell models do not fully recapitulate the complex splicing repertoire.

Patient-derived induced pluripotent stem cells (iPSCs) are scalable, tractable, and genetically faithful models, which are being increasingly utilized to understand myeloid pathobiology [23–30]. In a previous study, we established isogenic iPSC lines from MDS-*SF3B1* patients and identified a signature of splicing events associated with mutant *SF3B1* in hematopoietic stem and progenitor cells (HSPCs) [23]. Other work has further confirmed a splicing program paralleling the primary disease, supporting the use of *SF3B1*-mutant iPSCs as a physiologically relevant model [24].

In this study, we utilized RNA sequencing and splicing analysis to identify a previously unreported mis-splicing event affecting the ubiquitin-activating enzyme 1 (*UBA1*) gene specific to *SF3B1* mutations. *UBA1* is located on the X chromosome and encodes an essential multidomain protein that initiates the ubiquitination cascade, targeting proteins for degradation via the ubiquitin-proteasome system through the activation and transfer of ubiquitin to E2 enzymes. UBA1 is critical for cell survival, and is involved in protein folding and turnover, ER stress response, and DNA damage response [31–33]. Targeting the ubiquitin-proteasomal pathway, including UBA1, has been investigated for the treatment of hematologic malignancies and other cancers [34, 35]. We demonstrate that *SF3B1* mutation-associated *UBA1* mis-splicing (*UBA1*^ms^) leads to protein instability, resulting in reduced total UBA1 protein levels in *SF3B1*^K700E^ cell lines. This, in turn, sensitizes *SF3B1*-mutant cells to the UBA1 inhibitor TAK-243. We further confirm that *UBA1*^ms^ is unique to *SF3B1*-mutant patients in a large MDS cohort [36], and similarly enhances TAK-243 susceptibility in colony-forming assays using primary patient cells. Collectively, our findings establish *UBA1*^ms^ as a targetable therapeutic vulnerability in MDS-*SF3B1*.

## Materials and methods

### Patient-derived iPSCs

The generation and characterization of human iPSC lines MDS-22.44 (*SF3B1*^K700E^) and N-22.45 (*SF3B1*^WT^), from a female MDS patient with ring sideroblasts, single-lineage dysplasia and an isolated, heterozygous *SF3B1*^K700E^ variant, were previously described [23]. iPSCs were cultured on Matrigel hESC-Qualified Matrix (Corning) in mTeSR Plus (StemCell Technologies) and 1% Penicillin-Streptomycin (P/S; HyClone) and clump passaged with EZ-LiFT Stem Cell Passaging Reagent (Sigma-Aldrich). HSPCs were generated using STEMdiff Hematopoietic Kit (StemCell Technologies) and harvested on day 12. CD34^+^ cells were enriched using CD34 MicroBeads and positive selection with the autoMACS Pro Separator (Miltenyi Biotec). Erythroid differentiation and RNA sequencing can be found in Supplementary Methods.

### Cell culture

K562 human erythroleukemia cells with a heterozygous knock-in *SF3B1*^K700E^ mutation and the parental *SF3B1*^WT^ line were purchased from Horizon Discovery and cultured in RPMI 1640 Medium with 10% heat-inactivated fetal bovine serum (FBS; Gibco) and 1% P/S. Media was replaced every other day. HEK293T human embryonic kidney cells were maintained in DMEM, high glucose, GlutaMAX (Gibco), containing 10% newborn calf serum (Gibco) and 1% P/S. All cells were confirmed to be Mycoplasma-negative regularly.

### Primary sample collection and ethical approval

Bone marrow samples were obtained from three patients with MDS-*SF3B1* and two healthy normal bone marrow (NBM) donors at Karolinska University Hospital, Huddinge, Sweden. All source material was provided with written informed consent for research use, in accordance with the Declaration of Helsinki, and the study was approved by the Ethics Research Committee at Karolinska Institutet (2017/1090-31/4, 2022-03406-02 and 2024-03119-02).

### RNA splicing analysis

RNA sequencing data was assessed as previously described [8]. Briefly, differential splicing analysis was performed using rMATS v. 4.1.1 [37], with *p*-values calculated using the likelihood-ratio test (LRT) and adjusted with the Benjamini-Hochberg method. Sashimi plots for visualization were generated using ggsashimi v. 1.1.5 [38].

### *UBA1* expression plasmids

Human *UBA1* transcript variant 1 (WT) cDNA open reading frame (ORF) clone (accession number: NM_003334.4, CloneID: OHu24932) in a pcDNA3.1 + /C-(K)-DYK expression vector; pcDNA3.1 + C-eGFP (eGFP); and pcDNA3.1(+) (empty vector control) were purchased from GenScript. *UBA1*^ms^ cDNA ORF clone, including the 135 extra bases (MS), was subcloned into UBA1_OHu24932D_pcDNA3.1 + /C-(K)-DYK using GenScript’s gene synthesis and subcloning service. Plasmid transfection was achieved using Lipofectamine 2000 transfection reagent (Invitrogen) following manufacturer’s instructions. In brief, 2×10^5^ cells were seeded into 24-well culture plates in growth medium without P/S the day before transfection and cultured to ≥70% confluency. Lipofectamine complexes were prepared with 5 µg of *UBA1* WT, *UBA1* MS variant, pcDNA3.1, or eGFP plasmid in Opti-MEM (Gibco). RNA was collected after 48 h and protein at 72 h. See Supplementary Table 1 for additional details.

### Statistics

Statistical analysis was performed with Prism (v10.2, GraphPad). Data are shown as mean with standard error (SEM) unless otherwise noted. Numerical variables were compared using unpaired *t*-test, one-way analysis of variance (ANOVA), or two-way ANOVA, as indicated in the figure legends. For multiple test correction, *p*-values were adjusted by Šídák’s, Holm-Šídák’s, Tukey’s, or Dunnett’s multiple comparisons test, as specified.

### Supplementary methods

Additional details of the materials and methods are provided in Supplementary Methods.

## Results

### *SF3B1* mutation induces *UBA1*^ms^ in MDS-*SF3B1*

**Fig. 1:**
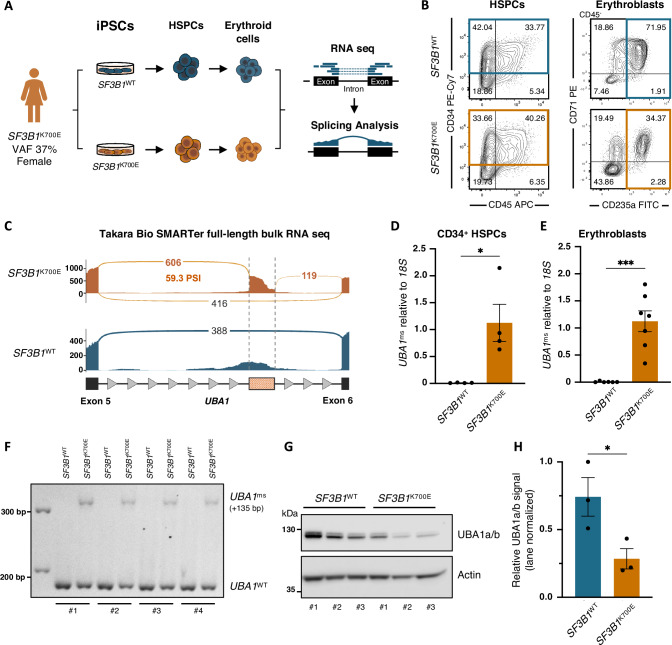
RNA sequencing reveals *UBA1*^ms^ in an iPSC model of MDS-*SF3B1.* **A** Origin of iPSC lines and experimental overview. **B** Representative flow cytometry diagrams of *SF3B1*^WT^ and *SF3B1*^K700E^ iPSC-derived cells after 12 days of hematopoietic differentiation (left) and another 14 days of erythroid culture (right). Colored gates indicate cells used in downstream analyses. **C** Sashimi plots of the mis-spliced region of *UBA1* in *SF3B1*^WT^ and *SF3B1*^K700E^ from total RNA sequencing of iPSC-derived GlyA^+^ erythroblasts (*n* = 1). Black, canonical splice junction counts; orange, mis-spliced junction counts. y-axis, absolute read counts. **D** qPCR analysis of *UBA1*^ms^ relative to *18S* in CD34^+^ HSPCs (*n* = 4) and **E** erythroblasts (*n*^WT^ = 6; *n*^K700E ^= 7) derived from *SF3B1*^WT^ and *SF3B1*^K700E^ iPSCs. Mean ± SEM relative expression. Unpaired *t*-test. **F** Agarose gel electrophoresis of the PCR-amplified exon 5-6 mis-spliced region of *UBA1* in iPSC-derived CD34^+^ cells (*n* = 4). The lower band corresponds to the PCR product of canonically spliced, and the upper band to mis-spliced *UBA1*. **G** Immunoblot analysis and **H** quantification of UBA1a/b protein levels in iPSC-derived CD34^+^ cells (*n* = 4). Actin was used as a loading control and relative signals were normalized by lane normalization factor. Mean ± SEM relative UBA1 signal intensity. Unpaired *t*-test with Holm-Šídák’s multiple comparisons test. *, *P* ≤ 0.05; ***, *P* ≤ 0.001. The numbers (#) below the blot indicate experimental repeats. VAF variant allele frequency, PSI percent spliced-in, RNA seq RNA sequencing, bp base pairs.

New treatment strategies for MDS-*SF3B1* require a deepened understanding of mis-splicing and the molecular consequences in sculpting the pathophysiology of MDS. Here, we employed isogenic iPSC lines originally generated from an MDS*-SF3B1* patient [23], harboring an isolated, heterozygous *SF3B1*^K700E^ mutation (Fig. 1A), the most recurrent variant among MDS*-SF3B1* cases [10]. To model MDS-*SF3B1* in vitro, we performed hematopoietic differentiation of wildtype (*SF3B1*^WT^) and mutant (*SF3B1*^K700E^) iPSCs for twelve days and analyzed surface expression of HSPC markers CD34 and CD45 by flow cytometry (Fig. 1B, left). Consistent with our previous observations, *SF3B1*^K700E^ HSPCs showed lower viability (supplementary Fig. 1A) but no defects in hematopoietic specification, with similar levels of CD34^+^ cells compared to *SF3B1*^WT^ (supplementary Fig. 1B). As before, *SF3B1*^K700E^ iPSCs exhibited reduced erythroid potential compared with *SF3B1*^WT^ cells (Fig. 1B, right) [23]. To examine *SF3B1* mutation-associated mis-splicing and uncover novel RNA mis-splicing events, we performed 5'-based full-length RNA sequencing of iPSC-derived erythroid cells to capture both immature and mature transcripts. Splicing analysis confirmed aberrant splicing of known genes, including *TMEM14C* and *MAP3K7*, in *SF3B1*^K700E^ but not *SF3B1*^WT^ cells (supplementary Fig. 1C), consistent with prior observations and findings in *SF3B1*-mutated patients [16, 24, 39]. Moreover, in this analysis we identified an RNA mis-splicing event of the *UBA1* transcript (59.3 percent spliced-in [PSI]) caused by the in-frame retention of an intronic region of 135 bases between exons 5 and 6 (Fig. 1C and supplementary Fig. 1D). *UBA1* encodes ubiquitin-activating enzyme E1, which catalyzes the first step of the ubiquitination pathway, and is necessary for protein homeostasis. Examination of this intron revealed an enrichment of cryptic SF3B1 binding sites, rendering it vulnerable to misrecognition (supplementary Fig. 1E and supplementary Table 4). We hypothesized that the non-conserved reads detected by RNA sequencing in *SF3B1*^WT^ resulted from intronic sequences in pre-mRNA, and utilized RT-qPCR for the *UBA1*^ms^ transcript to confirm that it is exclusive to *SF3B1*^K700E^ cells (supplementary Fig. 1F). As *SF3B1* mutations originate in the stem cell population [40], we assessed iPSC-derived CD34^+^ HSPCs and erythroid progenitors and observed that *SF3B1*^K700E^ but not *SF3B1*^WT^ cells expressed *UBA1*^ms^ (Fig. 1D, E), accounting for approximately 20% of total *UBA1* transcripts. To visualize the intronic sequence retention in CD34^+^ HSPCs, RT-PCR and gel electrophoresis of the *UBA1* exon 5-6 junction was performed (supplementary Fig. 1F). A second PCR product was generated in *SF3B1*-mutated cells, corresponding to the 135-base pair size difference resulting from mis-splicing, which was absent in *SF3B1*^WT^ (Fig. 1F and supplementary Fig. 1G). Next, we investigated whether *UBA1*^ms^ affects protein expression in iPSC-derived CD34^+^ HSPCs. *UBA1* encodes two main isoforms: nuclear UBA1a and cytosolic UBA1b. Immunoblotting against UBA1a/b revealed significantly reduced total UBA1 protein levels in *SF3B1*^K700E^ compared to *SF3B1*^WT^ cells (Fig. 1G, H). These results uncover previously unreported mis-splicing of *UBA1* in an in vitro model of MDS-*SF3B1* and suggest a decrease in UBA1 protein content in *SF3B1*-mutant cells.

### *UBA1*^ms^ decreases UBA1 protein in *SF3B1*^K700E^ cells

**Fig. 2:**
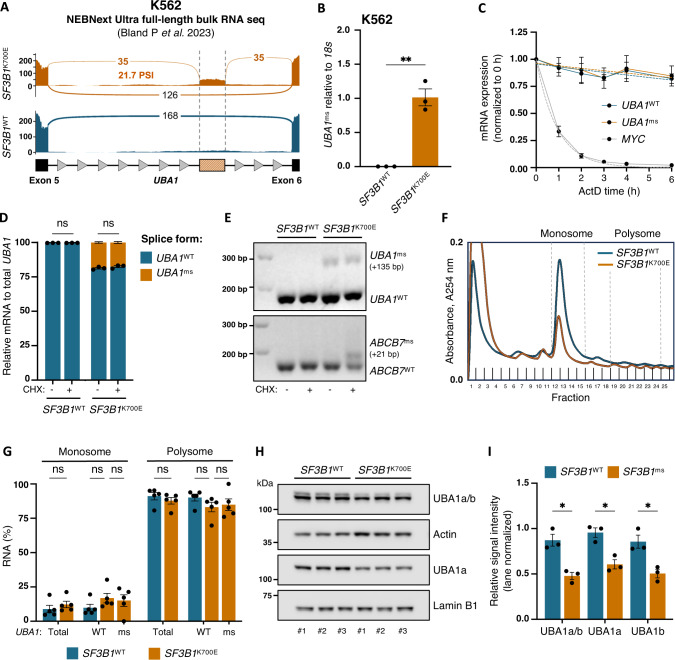
*UBA1*^ms^ leads to a decrease in UBA1 protein in *SF3B1*^K700E^ cells. **A** Sashimi plots of the *UBA1*^ms^ region from total RNA sequencing of *SF3B1*^WT^ and *SF3B1*^K700E^ K562 cells from previously published data [39]. **B** qPCR analysis of *UBA1*^ms^ relative to *18S* in *SF3B1*^WT^ and *SF3B1*^K700E^ K562 cells (*n* = 3). Mean ± SEM relative expression. Unpaired *t*-test. **C** qPCR analysis of *UBA1*^WT^ and *UBA1*^ms^ transcript levels in *SF3B1*^K700E^ K562 cells after treatment with actinomycin D (ActD) for the indicated time points (*n* = 3). Results were normalized to 0 h, and *MYC* was included as a fast-degrading transcript control. Mean ± SEM relative expression, One-phase decay nonlinear curve fit (dotted line). **D** qPCR analysis of *UBA1*^WT^ and *UBA1*^ms^ in *SF3B1*^WT^ and *SF3B1*^K700E^ K562 cells after treatment with cycloheximide (CHX) for 4 h (*n* = 3). The fraction of *UBA1* splice forms is shown within the bars. Mean ± SEM relative expression. Two-way ANOVA with Tukey’s multiple comparisons test. **E** Agarose gel electrophoresis of the PCR-amplified mis-spliced region of *UBA1* (top) and *ABCB7* (bottom) in *SF3B1*^WT^ and *SF3B1*^K700E^ K562 cells. The lower bands correspond to the PCR product of canonically spliced RNA, and the upper bands to mis-spliced RNA. Representative image from three experimental repeats. **F** Representative polysome profiles of *SF3B1*^WT^ and *SF3B1*^K700E^ K562 cells recorded at 254 nm. Pooled monosome and polysome fractions are indicated. **G** qPCR analysis of total *UBA1*, *UBA1*^WT^, and *UBA1*^ms^ RNA transcript distribution in monosome and polysome fractions from *SF3B1*^WT^ and *SF3B1*^K700E^ K562 cells (*n* = 5). Housekeeping gene transcript distribution can be found in Supplementary Fig. 2C. Mean ± SEM fraction. Two-way ANOVA with Tukey’s multiple comparisons test. **H** Immunoblot analysis and **I** quantification of UBA1 isoforms in whole cell lysates from *SF3B1*^WT^ and *SF3B1*^K700E^ K562 cells (*n* = 3). Actin was used as a loading control for total UBA1 and UBA1b; Lamin B1 was used as a loading control for nuclear UBA1a, and relative signals were normalized by lane normalization. Mean ± SEM relative signal intensity. Unpaired *t*-test with Holm-Šídák’s multiple comparisons test. *, *P* ≤ 0.05; **, *P* ≤ 0.01; ns not significant.

To investigate whether *UBA1*^ms^ is a specific consequence of *SF3B1* mutation, we utilized the *SF3B1*^K700E^ K562 human erythroleukemia cell line. Similar to iPSCs, K562 cells harboring *SF3B1* mutation recapitulate the splicing landscape of primary MDS [17, 19]. First, analysis of a recently published full-length K562 RNA sequencing dataset also displayed the *UBA1*^ms^ event in *SF3B1*^K700E^ but not in *SF3B1*^WT^ K562 cells, which was not reported by the authors (Fig. 2A) [41]. As before, RT-qPCR of *UBA1*^ms^ and visualization of the retained intron by gel electrophoresis confirmed that *UBA1*^ms^ is specific to *SF3B1*^K700E^ (Fig. 2B and supplementary Fig. 2A). RNA mis-splicing induced by mutant *SF3B1* can trigger nonsense-mediated decay (NMD) when a premature termination codon is introduced, disrupting ribosome dynamics [13]. However, in this case, the cryptic exon resulting from *UBA1*^ms^ does not contain an in-frame premature stop codon. Experiments using actinomycin D (ActD) to inhibit transcription in *SF3B1*^K700E^ K562 cells confirmed that *UBA1*^ms^ does not impair mRNA stability. RT-qPCR analysis revealed that *UBA1*^ms^, like canonically spliced *UBA1* (*UBA1*^WT^), remains stable, whereas short-lived *MYC* mRNA undergoes rapid degradation (Fig. 2C). Additionally, inhibition of NMD with cycloheximide (CHX) did not result in an increase in *UBA1*^ms^ (Fig. 2D, E). In contrast, the well-characterized NMD target resulting from mis-splicing of *ABCB7* was only detectable upon NMD inhibition, as shown by gel electrophoresis (Fig. 2E), consistent with previous findings [39].

To determine whether *UBA1*^ms^ affects ribosome recruitment, we performed polysome profiling and RT-qPCR on monosome and polysome fractions from *SF3B1*^K700E^ and *SF3B1*^WT^ K562 cells (Fig. 2F). *UBA1* transcripts were predominantly associated with the polysome fraction, indicating active translation irrespective of splice variant, unlike NMD targets, which are typically depleted from polysomes [42, 43]. Moreover, the distribution of *UBA1* transcripts across fractions remained consistent between mutant and wild-type cells, reflecting patterns observed in housekeeping genes (Fig. 2G and supplementary Fig. 2B). However, similar to our findings in iPSC-derived CD34^+^ cells, total UBA1 protein levels in *SF3B1*^K700E^ K562 cells were significantly reduced compared to *SF3B1*^WT^ cells, owing to lower levels of both UBA1a and UBA1b isoforms (Fig. 2H, I). In summary, these data validate *SF3B1* mutation-specific *UBA1*^ms^ in an independent cell model and demonstrate decreased total UBA1 protein levels in mutant cells despite stable transcript levels and intact ribosome assembly.

### Mis-splicing of *UBA1* compromises protein stability

To determine the consequence of *UBA1*^ms^ on protein stability, we first modeled its structure in silico. AlphaFold2 was used to predict protein folding of the 1058 amino acid UBA1a isoform from full-length *UBA1* (*UBA1*^WT^) cDNA (ENST00000377351.8) and *UBA1*^ms^ including the predicted 45 amino acid sequence (supplementary Fig. 1D). The insertion resulted in low sequence coverage, low predicted local distance difference test score (< 50%), and high predicted aligned error (supplementary Fig. 3A–C). Consequently, the model failed to confidently predict folding of amino acid residues from *UBA1*^ms^ (supplementary Fig. 3D).

**Fig. 3:**
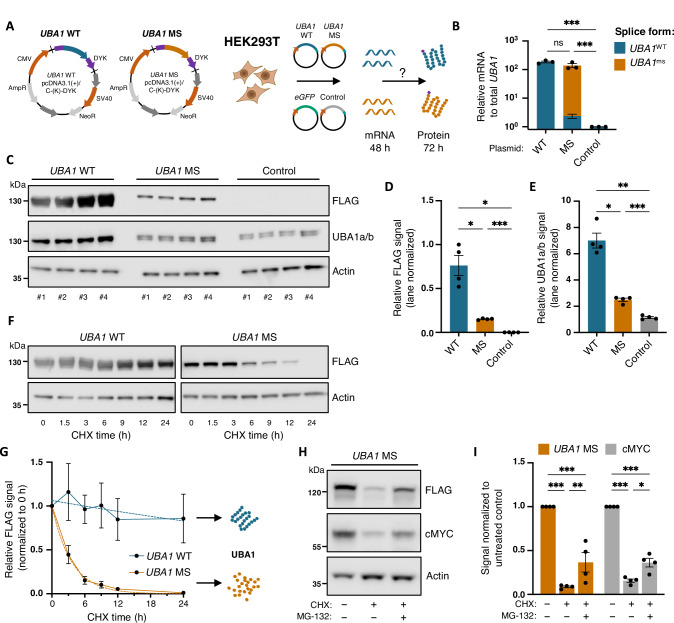
*SF3B1*^K700E^-specific *UBA1*^ms^ compromises protein stability. **A** Maps for ORF cDNA in pcDNA3.1 + /C-(K)-DYK plasmids for *UBA1* transcript variant 1 (WT) and *UBA1*^ms^ (MS) and experimental design for evaluation of *UBA1*^ms^ in HEK293T cells. **B** qPCR analysis of *UBA1* transcript levels in HEK293T cells 48 h following transfection with WT or MS relative to pcDNA3.1 control transfected cells. The fraction of *UBA1* splice forms is shown within bars (*n* = 3). Mean ± SEM relative fold change. Two-way ANOVA with Tukey’s multiple comparisons test. **C** Immunoblot analysis and quantification of **D** FLAG-tagged and **E** UBA1a/b protein levels in HEK293T cells 72 h after transfection with WT, MS or control. Actin was used as a loading control and the highest relative FLAG and lowest relative UBA1a/b signals were used to normalize results (*n* = 4). Mean ± SEM relative signal intensity. Welch ANOVA with Dunnett’s multiple comparisons test. **F** Representative immunoblot analysis and **G** quantification of FLAG-tagged protein levels in HEK293T cells 72 h post-transfection with WT, MS, or control plasmids and treatment with 50 µg/ml cycloheximide (CHX) for the indicated time points (*n* = 3). Actin was used as a loading control and signals were normalized to relative signals at 0 h for each group. Mean ± SEM relative signal intensity, interpolation of a one-phase decay non-linear regression curve (dotted line). **H** Representative immunoblot analysis and **I** quantification of FLAG-tagged UBA1^ms^ and cMYC protein levels in HEK293T cells 72 h post-transfection with the MS plasmid and treatment with 50 µg/ml cycloheximide (CHX) and 10 µM MG-132 for 6 h (*n* = 4). Actin was used as a loading control and signals were normalized to untreated controls. Mean ± SEM relative signal intensity. Two-way ANOVA with Tukey’s multiple comparisons test. *, *P* ≤ 0.05; **, *P* ≤ 0.01; ***, *P* ≤ 0.001; ns not significant.

To experimentally test whether *UBA1*^ms^ compromises protein stability, we designed expression plasmids for *UBA1*^WT^ (WT) and *UBA1*^ms^ (MS) fused to a C-terminal FLAG tag for transfection of HEK293T cells and subsequent assessment of recombinant protein levels (Fig. 3A and supplementary Fig. 4A). Total *UBA1* transcript levels, measured by RT-qPCR after 48 h, were elevated in cells transfected with WT or MS plasmids compared to control, and expression of *UBA1*^ms^ was exclusive to *UBA1* MS-transfected cells (Fig. 3B). We next validated high transfection efficiency and cell viability at 72 h post-transfection (supplementary Fig. 4B, C), followed by immunoblotting to examine recombinant UBA1 translation. These results revealed one FLAG band corresponding to the predicted product from the ORF. Interestingly, FLAG-tagged UBA1 protein levels were significantly lower in MS- compared to WT-transfected cells, which was recapitulated in UBA1a/b expression levels (Fig. 3C–E). To determine whether this reduction in UBA1 levels result from impaired protein stability, we performed CHX chase assays of cells transfected with *UBA1* WT or MS plasmid. Immunoblotting against FLAG at the indicated time points revealed that UBA1^WT^ protein levels remained stable, consistent with the reported half-life exceeding 100 h [44], whereas UBA1^ms^ protein displayed rapid decay, with a calculated half-life of 2.5 h (Fig. 3F, G**)**. We confirmed proteasomal degradation of UBA1^ms^ protein by co-treating transfected cells with CHX and the proteasome inhibitor MG-132. This partially restored FLAG-tagged UBA1^ms^ protein levels, mirroring the stabilization of the short-lived endogenous cMYC protein, which served as a positive control (Fig. 3H, I). Together, these data show that UBA1 protein produced from mis-spliced mRNA is unstable and degraded, explaining the reduction in total UBA1 levels in models of mutant *SF3B1*.

### *SF3B1* mutation sensitizes K562 cells to UBA1 inhibition

**Fig. 4:**
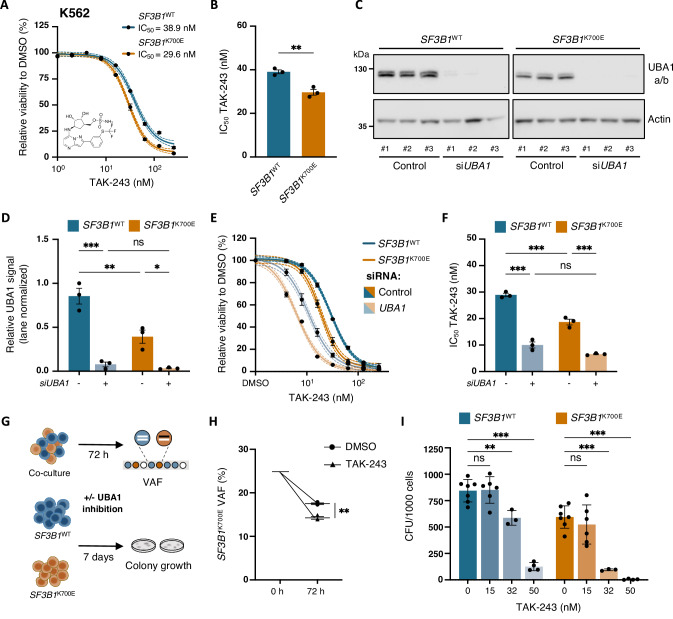
*SF3B1* mutation sensitizes K562 cells to UBA1 inhibition. **A** Dose-response curves of *SF3B1*^WT^ and *SF3B1*^K700E^ K562 cells treated with TAK-243 or DMSO for 72 h (*n* = 3). Chemical structure of the small-molecule UBA1 inhibitor TAK-243 (MLN7243) displayed under the curve. Data points represent mean ± SEM live cell fractions, normalized to control-treated samples. Interpolated sigmoidal, four parameter logistic (4PL), standard curves of mean (solid line) and 95% confidence interval (dotted line). Live cells were defined as Aqua/Apotracker Green double negative singlets, assessed by flow cytometry (see supplementary Fig. 5A for gating strategy). IC_50_ of TAK-243 are quantified in **B**. Mean ± SEM nM TAK-243. Unpaired *t*-test. **C** Immunoblot analysis and **D** quantification of UBA1a/b protein levels in *SF3B1*^WT^ and *SF3B1*^K700E^ K562 cells 72 h after transfection with negative control siRNA (control) or *UBA1* siRNA (si*UBA1*). Actin was used as a loading control and results were normalized to the highest relative signal. Mean ± SEM relative UBA1a/b signals. Two-way ANOVA with Tukey’s multiple comparisons test. **E** Dose-response curves of *SF3B1*^WT^ and *SF3B1*^K700E^ K562 cells 72 h after transfection with negative control siRNA (control) or *UBA1* siRNA (si*UBA1*), treated with TAK-243 or DMSO for 72 h (*n* = 3). Data points represent mean ± SEM live cell fractions, normalized to control-treated samples. Interpolated sigmoidal, 4PL, standard curves of mean (solid line) and 95% confidence interval (dotted line). Live cells were defined as Aqua/Apotracker Green double negative singlets, assessed by flow cytometry. IC_50_ of TAK-243 are quantified in **F**. Mean ± SEM nM TAK-243. Two-way ANOVA with Tukey’s multiple comparisons test. **G** Experimental strategy to assess the effect of targeted UBA1 inhibition by TAK-243 treatment on *SF3B1*^WT^ and *SF3B1*^K700E^ cells. **H ***SF3B1*^K700E^ VAF in co-cultures of 1:1 *SF3B1*^WT^ and *SF3B1*^K700E^ K562 cells after 72 h of treatment with 50 nM TAK-243 or DMSO, as determined by ddPCR (*n* = 3). Mean ± SEM. Unpaired *t*-test. **I** CFU counts per 1000 seeded *SF3B1*^WT^ and *SF3B1*^K700E^ K562 cells treated with indicated concentrations of TAK-243 for 7 days (*n*^DMSO^ = 7, *n*^15 nM^ = 5, *n*^32 nM^ = 3; *n*^50 nM^ = 4). Mean ± SEM. Two-way ANOVA with Dunnett’s multiple comparisons test. *, *P* ≤ 0.05; **, *P* ≤ 0.01; ***, *P* ≤ 0.001; ns not significant.

UBA1 plays a critical role in cellular proteostasis and loss of *UBA1* induces cell death [32, 33, 45], indicating a minimum level of UBA1 activity is required for cell survival. We hypothesized that a reduction in UBA1 protein content in *SF3B1*-mutant cells would confer higher sensitivity to targeted UBA1 inhibition compared to *SF3B1*^WT^ cells. To test this, the selective UBA1 inhibitor TAK-243 [34] (Fig. 4A) was employed to investigate the effect of UBA1 inhibition on cell viability, clonal competition, and colony-forming unit (CFU) potential in *SF3B1*-mutated cells. First, we treated *SF3B1*^WT^ and *SF3B1*^K700E^ K562 cells with increasing concentrations of TAK-243 and conducted flow cytometry and luminescence-based assays after 72 h to determine IC_50_. TAK-243 induced apoptosis in a concentration-dependent manner and, importantly, with significantly higher sensitivity in *SF3B1*^K700E^ K562 cells (Fig. 4A, B and supplementary Fig. 5A–D). Additionally, TAK-243 reduced K48-linked ubiquitin levels in a dose-dependent manner and led to a marked increase in apoptosis-associated cleaved PARP1 and cleaved Caspase-3 in *SF3B1*^K700E^ compared to  wild-type (supplementary Fig. 5E–J). We confirmed UBA1 specificity, as mutated cells did not exhibit differential sensitivity to other commonly used drugs to treat MDS and AML, cytarabine, daunorubicin, pladienolide B, and venetoclax, compared to *SF3B1*^WT^ cells (supplementary Fig. 6A–D). Furthermore, small interfering RNA (siRNA)-mediated knockdown followed by TAK-243 exposure significantly reduced the IC_50_ compared to control-transfected *SF3B1*^WT^ and *SF3B1*^K700E^ cells (Fig. 4C–F supplementary Fig. 6E), further supporting the selective vulnerability of *SF3B1*-mutant cells to UBA1 inhibition.

We next assessed the sensitivity of *SF3B1*-mutated K562 cells to TAK-243 through competitive culture between wild-type and mutant cells with short-term, 72-h TAK-243 treatment (quantification of variant allele frequency [VAF] by droplet digital PCR [ddPCR]), as well as CFU assays with continuous TAK-243 exposure for seven days (Fig. 4G). Indeed, TAK-243 significantly reduced *SF3B1*^K700E^ mutational burden in co-cultures, albeit with modest effect (Fig. 4H), likely attributed to the proliferative advantage of *SF3B1*^WT^ cells in vitro [23] (supplementary Fig. 6F), as supported by a reduction in *SF3B1*^K700E^ VAF after 72 h in control conditions. In addition, *SF3B1*^K700E^ cells exhibited lower overall CFU potential compared to *SF3B1*^WT^ (Fig. 4I), consistent with previous findings [23]. UBA1 inhibition affected CFU potential in both *SF3B1*^K700E^ and *SF3B1*^WT^ cells in a concentration-dependent manner. TAK-243 treatment with 32 nM or 50 nM induced a significantly differential effect on CFU potential, but 50 nM severely impaired wild-type cells (supplementary Fig. 6G). Notably, the calculated IC_50_ values reflected the enhanced sensitivity of *SF3B1*-mutated cells to UBA1 inhibition compared to *SF3B1*^WT^ (supplementary Fig. 6G). Together, these results define a therapeutic window in which *SF3B1*^K700E^ cells, but not *SF3B1*^WT^ cells, are selectively sensitized to UBA1 inhibition, providing an opportunity to diminish the *SF3B1*-mutant population while preserving wild-type clones.

### *UBA1*^ms^ in MDS-*SF3B1* patients confers sensitivity to targeted UBA1 inhibition

**Fig. 5:**
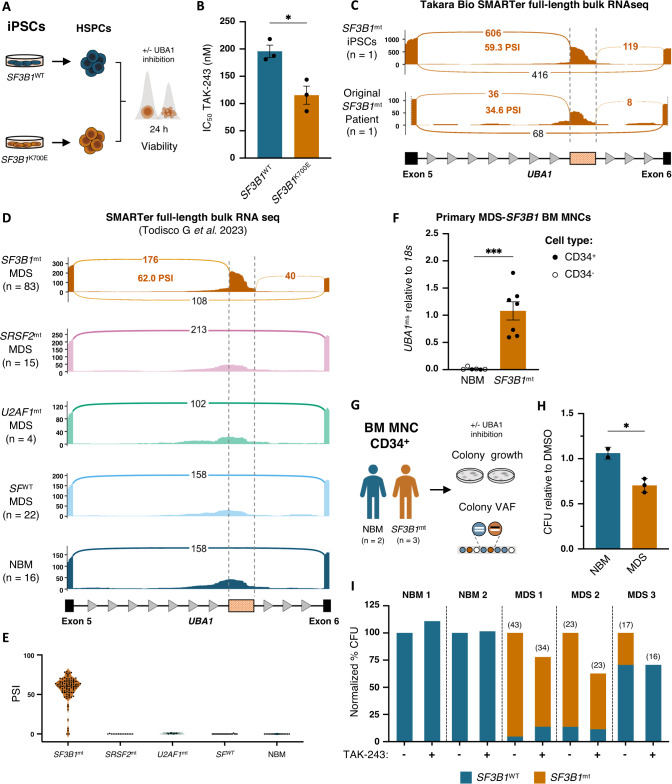
*UBA1*^ms^ in MDS-*SF3B1* patients confers sensitivity to UBA1 inhibition. **A** Experimental strategy to assess the effect of UBA1 inhibition by TAK-243 on the viability of HSPCs derived from *SF3B1*^WT^ and *SF3B1*^K700E^ iPSCs. **B** IC_50_ of TAK-243 in *SF3B1*^WT^ and *SF3B1*^K700E^ iPSC-derived HSPCs treated with TAK-243 or DMSO for 24 h (*n* = 3), quantified from dose-response curves in supplementary Fig. 7B. Mean ± SEM. Unpaired *t*-test. **C** Sashimi plots of the mis-spliced region in *UBA1* from total RNA sequencing of *SF3B1*^K700E^ iPSC-derived erythroblasts and primary CD34^+^ BM MNCs from the original MDS-*SF3B1* patient. **D** Sashimi plots of read counts of the mis-spliced region in *UBA1* in MDS-RS patients and healthy donors, grouped by splicing factor mutation status, from our previously published data [36] (*n*^*SF3B1*^ = 83, *n*^*SRSF2*^ = 15, *n*^*U2AF1*^ = 4, *n*^SFWT^ = 22, *n*^NBM^ = 16). **E** Violin plots of *UBA1* intron 5 mis-splicing PSI from total RNA sequencing of CD34^+^ BM MNCs from the same patient cohort, organized by splicing factor mutation. **F** qPCR analysis of *UBA1*^ms^ relative to *18S* in CD34^+^ (filled circles) or CD34^-^ (empty circles) cells from primary BM MNCs of healthy donors (NBM; *n* = 6) and *SF3B1*-mutated MDS patients (*SF3B1*^mt^; *n* = 7). Mean ± SEM relative expression. Unpaired *t*-test **G** Experimental strategy to assess the effect of UBA1 inhibition on colony growth and composition in CD34^+^-enriched BM MNCs from MDS-*SF3B1* patients and healthy controls. **H** Effect of UBA1 inhibition on CFU counts relative to DMSO and **I** frequency of *SF3B1*^WT^ and *SF3B1*^mt^ colonies relative to total CFU counts from MDS patient (*n* = 3) or healthy control (*n* = 2) cells treated with 32 nM TAK-243 or DMSO for 14 days. Numbers within brackets indicate colonies assessed by ddPCR. Mean ± SEM. Unpaired *t*-test. *, *P* ≤ 0.05; ***, *P* ≤ 0.001; ns not significant. *SF3B1*^mt^* SF3B1-*mutated, MDS-RS MDS with ring sideroblasts, NBM normal bone marrow from healthy donors.

We further probed the increased sensitivity of *SF3B1*-mutated cells to UBA1 inhibition by treating patient iPSC-HSPCs with increasing concentrations of TAK-243 over 24 h and measuring cell viability by flow cytometry (Fig. 5A and supplementary Fig. 7A). As before, TAK-243 was more potent at inducing cell death in *SF3B1*^K700E^ iPSC-HSPCs compared to *SF3B1*^WT^ cells (supplementary Fig. 7B), resulting in a significantly lower IC_50_ in *SF3B1*^K700E^ HSPCs (Fig. 5B). Importantly, splicing analysis of RNA sequencing data from bone marrow mononuclear cells (BM MNC) of the original MDS-*SF3B1* patient used for the iPSC reprogramming [23] confirmed that *UBA1*^ms^ is a common event in both the mutant cell line and the primary cells (Fig. 5C). To evaluate the clinical relevance of our findings and to verify how *UBA1*^ms^ is recapitulated in patients, we performed splicing analysis in our previously reported MDS cohort [36]. Specifically, we examined the mis-splicing event in 124 patients with MDS with ring sideroblasts and a VAF ≥ 20% (*SF3B1*^mt^, *n* = 83; *SRSF2*^mt^, *n* = 15; *U2AF1*^mt^, *n* = 4; and splicing factor^WT^, *n* = 22) compared to healthy donors (*n* = 16) using full-length total RNA sequencing data from CD34^+^ BM MNCs. *UBA1*^ms^ was abundant (57.31 ± 1.78 PSI) and exclusive to *SF3B1*-mutated patients but not present in other splicing factor-mutated or wild-type cases nor in healthy donors (Fig. 5D, E). To validate these results, we assessed an independent long-read single-cell RNA sequencing dataset [46]. Although the *UBA1*^ms^ event was not originally reported, splicing analysis confirmed it to be exclusive to *SF3B1*-mutated cases (supplementary Fig. 8A). This transcriptomic data also enabled verification that *UBA1*^ms^ results in a full-length transcript. Total *UBA1* transcript levels were not significantly different between CD34^+^ cells from sex-matched MDS-*SF3B1* cases and healthy donors in our cohort. However, *UBA1* transcript levels were overall higher in female compared to male individuals (supplementary Fig. 8B), likely due to an escape from X inactivation [47].

Among the *SF3B1* mutation cohort, *SF3B1*^K700E^ patients (50 of 83) exhibited similar levels of *UBA1*^ms^ compared to patients with other *SF3B1* variants (28 of 33) (Supplementary Fig. 8C–E). Additionally, mis-splicing levels were consistent across *SF3B1*^K700E^ VAFs (Supplementary Fig. 8F). We confirmed the presence of *UBA1*^ms^ in HSPCs from MDS-*SF3B1* patients and its absence in NBM, by RT-qPCR and RT-PCR, using primary CD34^+^ cells (and CD34^-^ cells when cell count was limited, as indicated) (Fig. 5F and supplementary Fig. 8G).

Given the abundance of *UBA1*^ms^ in MDS-*SF3B1* patients and the instability of UBA1^ms^ protein, we hypothesized a partial loss of functional UBA1 protein that could be exploited therapeutically. To assess whether targeted UBA1 inhibition through TAK-243 in primary HSPCs from MDS patients would selectively reduce *SF3B1*-mutant cells, as observed with K562 cells, we performed CFU assays with CD34^+^ BM MNCs from sex-matched *SF3B1*-mutated MDS patients and healthy donors (Fig. 5G). Indeed, TAK-243 exposure significantly reduced the CFU potential relative to DMSO of cells from MDS patients compared to healthy donors (Fig. 5H). Single-colony genotyping by ddPCR confirmed that the treatment effect in *SF3B1*-mutated patient cells largely resulted from a reduction of mutated colonies, whereas CFU formation from residual wild-type cells was less affected (Fig. 5I). Taken together, we confirm extensive *UBA1*^ms^ exclusive to *SF3B1*-mutated patients, conferring a sensitivity of mutant cells to targeted inhibition of UBA1 with TAK-243 in both iPSC-derived and primary HSPCs.

## Discussion

A detailed understanding of mis-splicing and ensuing molecular consequences in splicing factor-mutated MDS is key for the development of improved therapies. Advances in single-cell and integrative genomics have empowered the discovery of novel MDS-*SF3B1* disease mechanisms, presenting potential therapeutic modalities [8, 19, 46]. Here, we leveraged in vitro models of MDS-*SF3B1* and full-length RNA sequencing data with unsupervised analysis to identify *UBA1* as a target of mis-splicing with adverse effects on UBA1 protein levels. This finding was corroborated by MDS patient cohort analyses, in which *UBA1*^ms^ was exclusively present in *SF3B1-*mutant individuals. Our study underscores the relevance of iPSCs as a valuable tool in hematological research and highlights an *SF3B1*-driven RNA mis-splicing event with therapeutic implications.

*SF3B1* is the most frequently mutated component of the spliceosome machinery, associated with widespread disruption of RNA splicing [3, 8, 9, 13, 16, 18, 21]. Previously characterized *SF3B1*-mutant mis-spliced transcripts typically introduce a premature stop codon and are therefore targeted for NMD. This is not the case for *UBA1*^ms^, which encodes a 45-amino-acid insertion within the inactive adenylation domain of UBA1. Consistent with this, we confirmed mRNA stability, absence of NMD, and successful engagement of *UBA1*^ms^ mRNA with the translation machinery. Although a recent study reported decreased *UBA1* RNA and protein expression in splicing gene–mutant MDS, albeit without a link to RNA mis-splicing of *UBA1* [48], our data do not show a difference in *UBA1* transcript expression levels between *SF3B1*-mutant individuals and healthy donors. Instead, we attribute the reduction of total UBA1 to the instability of the UBA1^ms^ protein product, as evidenced by its short half-life in our in vitro model compared to UBA1^WT^ [44]. Consequently, the rapid degradation of UBA1^ms^ prevents its detection by immunoblotting, leaving only the canonically spliced UBA1 isoforms visible in *SF3B1*-mutant cells. Previous work by us and others has described stage-specific, pervasive mis-splicing due to *SF3B1* mutation and induction of pro-survival mechanisms in mutated cells [8, 46]. In contrast, lower UBA1 protein levels due to *UBA1*^ms^ would likely challenge mutant cell viability as UBA1 is essential for protein homeostasis and cell survival [32, 33]. Curiously, a recent study reported an adaptive stress response in cells with a partial reduction in UBA1 activity that confers cellular resilience [49], which would be in line with the clonal advantage of *SF3B1*-mutated hematopoietic stem cells.

Somatic, heterozygous mutations in the *UBA1* gene have recently gained attention in VEXAS (vacuoles, E1 enzyme, X-linked, autoinflammatory, somatic) syndrome [50]. In this case, UBA1b expression is specifically lost and the accumulation of a catalytically impaired isoform leads to disruption of the ubiquitin-proteasome system. In contrast, mis-splicing of *UBA1*, driven by cryptic SF3B1 splice site recognition, impacts both isoforms, leading to reduced levels of UBA1a and UBA1b without causing a complete loss of function. The shared convergence of *UBA1*, as a target of mis-splicing in MDS-*SF3B1* and somatic mutation in VEXAS, leads to two distinct clonal disorders. VEXAS manifests with inflammatory and hematological symptoms, with approximately half of affected patients also presenting with MDS [51]; however, MDS-*SF3B1* individuals typically exhibit a non-inflammatory phenotype and patient cells have been associated with a low inflammatory profile compared to other low-risk MDS subgroups [52]. Despite the different clinical phenotypes, it is tempting to speculate that exploiting UBA1 dysfunction might be a plausible treatment strategy in both myeloid disorders. Indeed, a recent study of a VEXAS cell model showed selective susceptibility to UBA1 modulation with the specific inhibitor TAK-243 [45], also used in this study. In line with this report, our data indicate a differential response of *SF3B1*-mutated cells to TAK-243 compared to *SF3B1*^WT^, potentially supporting the specificity and applicability of UBA1 inhibition in the development of pharmacological intervention for MDS-*SF3B1*. Utilizing a combination of cell models, HSPCs derived from iPSCs, and primary cells from MDS patients, we observed cell type-specific and concentration-dependent responses to UBA1 inhibition. While relative differences in sensitivity were modest, mutant cells consistently presented an increased susceptibility to UBA1 inhibition compared with wild-type cells.

*SF3B1* mutations are a favorable prognostic factor in MDS. Nevertheless, patients frequently develop transfusion-dependency, and allogenic stem cell transplantation remains the only curative option. While current treatment regimens primarily aim to manage the anemia, they do not specifically target the mutant HSPC compartment. A therapeutic strategy which depletes mutated cells in the bone marrow necessitates the presence of wild-type HSPCs to replenish the exhausted cells. Our results suggest that TAK-243 selectively reduces mutated cells while preserving the residual wild-type cells. Although TAK-243 did not completely eradicate mutant cells in CFU assays, it may present an opportunity for a mild, minimally toxic pharmacological intervention to decrease mutational burden and delay allogenic stem cell transplantation. Future work is warranted exploring combination treatments with other targets of *SF3B1* mutations. Given a role for UBA1 in the DNA damage response, and an observed increase in DNA damage in *SF3B1*-mutant cells, exploiting synthetic lethality, such as combining PARP or ATR inhibitors with TAK-243, could present a strategy to selectively eliminate mutant clones [41, 53]. Interestingly, TAK-243 has been shown to induce apoptosis in several hematological cancer models, including acute myeloid leukemia [35, 54, 55] and is currently being evaluated in a clinical trial for intermediate-2 or high-risk refractory MDS and leukemias (NCT03816319). Results from this trial will provide valuable insights into the dosing and toxicity profiles, informing decision-making for future clinical utility for MDS-*SF3B1*. In conclusion, we identify *UBA1* as a previously unrecognized target of *SF3B1*-mutant mis-splicing, which introduces a specific vulnerability of *SF3B1*-mutated cells to directed UBA1 inhibition.

## Supplementary information

Supplementary Methods and Figures

## Data Availability

The raw data for the RNA sequencing and corresponding metadata and intermediate analysis has been deposited as SND-ID: 2024-128 (Bulk RNA sequencing of erythroblasts from a pair of *SF3B1*-mutated and *SF3B1*-wildtype induced pluripotent stem cell (iPSC) lines) on the Swedish National Data Service’s research data repository. These data have been uploaded with restricted access due to their personally identifiable information status but are accessible upon reasonable request through SND at: 10.48723/3hs1-0v44. Other raw data are available from the corresponding author upon request.
