# Supplementary material for: SF3B1-mutant models of RNA mis-splicing uncover UBA1 as a therapeutic target in myelodysplastic neoplasms
Source: Leukemia. 2025 Aug 26;39(11):2801–11. doi: 10.1038/s41375-025-02740-1 (PMC12589102; doi:10.1038/s41375-025-02740-1)
Supplement: Supplementary file 1 — Supplementary Methods and Figures [file 41375_2025_2740_MOESM1_ESM.pdf]

Supplementary Data

Supplementary Methods

Erythroid differentiation and RNA sequencing

Hematopoietic differentiation was adapted from Matsubara *et al*. (PMID: 30948156): iPSCs were seeded as clumps and differentiated in Essential 8 Medium (Gibco), 1% P/S, 80 ng/ml VEGF, 80 ng/ml BMP4, and 2 µM CHIR-99021 from day 0; Essential 6 Medium (Gibco), 1% P/S, 80 ng/ml VEGF, 50 ng/ml SCF, and 2 µM SB431542 from day 2; and Stemline II (Sigma-Aldrich), 1% P/S, 50 ng/ml SCF, 50 ng/ml FLT3L, and 50 ng/ml IL3 from day 4-13 with media changes every two days. For erythroid specification, cells were cultured for 14 days in StemPro-34 SFM with 1% Bovine Albumin Fraction V (Gibco), 1% P/S, 2 mM L-Glutamine, 3.5 µM 1-Thioglycerol, 150 µg/ml holo-Transferrin (Sigma-Aldrich), 2 U/ml Erythropoietin (Retacrit, Pfizer), 50 ng/ml SCF and 50 ng/ml IL3, which was omitted from day 8, with media changes every other day. Cytokines were purchased from PeproTech; and CHIR99021 and SB431542 from StemCell Technologies. Glycophorin A$^+$ cells were enriched using CD235a Microbeads and positive selection with autoMACS Pro Separator (Miltenyi Biotec). Automated RNA extraction was performed using QIAcube Connect with RNeasy Micro Kit (Qiagen). Full-length bulk RNA sequencing and downstream analysis were performed, as previously described.[8]

Flow Cytometry

Flow cytometry experiments were performed on a BD LSRFortessa and analyzed with FlowJo v10.10 (BD Biosciences). Cells were stained for 30 min on ice using CD34-Pe-Cy7 and CD45-APC for HSPCs or CD45-APC, CD71-PE and GlyA-FITC for erythroblasts with 1:200 Aqua Live/Dead viability stain (Thermo Scientific). All steps were performed in FACS Buffer (PBS + 2% FBS+2 mM EDTA) and cells were fixed in 2% PFA prior to analysis. Antibody specifics are listed in Supplementary Table 2.

Primer Design

For splice form quantification, primer design was adapted from (PMID: 26979160) to generate primer pairs spanning the normal splice junction (canonical), priming within the mis-spliced sequence (variant) and upstream of the splice site (external control) using Primer-BLAST primer design tool (https://www.ncbi.nlm.nih.gov/tools/primer-

blast/). Primers had to meet default requirements with the following modifications: Primers must span an exon-exon junction with a product range between 70-200 nucleotides, a GC content from 40-60%, and include a GC clamp. Primer pair specificity was analyzed using the *Homo sapiens* Refseq mRNA database. Primers had at least four total mismatches, including at least three within the last four 3' base pairs and a maximum amplicon size. Other primers were generated using PrimerQuest tool (Integrated DNA Technologies) and checked for specificity using Primer-BLAST.

Quantitative reverse transcription PCR (RT-qPCR)

Cells were lysed in 350 µl Buffer RLT Plus (Qiagen) containing 40 mM 1,4-Dithiothreitol (DTT; Sigma-Aldrich). Automated RNA extraction was performed using QIAcube Connect with RNeasy Mini Kit (Qiagen) and RNA yield and purity measured by Nanodrop 2000 Spectrophotometer (Thermo Scientific). cDNA was generated using Maxima First Strand cDNA Synthesis Kit for RT-qPCR (Thermo Scientific) from 200 ng RNA. 2 or 4 ng cDNA was analyzed in triplicate by two-step RT-qPCR using PowerUp SYBR Green and StepOnePlus Real-Time PCR System (Applied Biosystems). All experiments included non-template negative controls. *UBA1* splice variant was quantified by fold change expression relative to *18S*, and total *UBA1* external control, where applicable. Primer details are listed in Supplementary Table 1.

PCR detection of splice variants

For visualization of the *UBA1*^{ms} and *ABCB7*^{ms} region, specific primers flanking the exon 5-6 and exon 8-9 junction were used, respectively (Supplementary Table 1) for PCR amplification of cDNA from the indicated samples using Phusion Flash PCR Master Mix (Thermo Scientific). PCR products were separated by electrophoresis on a 3.5% agarose gel (Invitrogen) using GeneRuler 100 bp Plus DNA ladder (Invitrogen) and bands visualized on an AlphaImager HP gel imager (Proteinsimple).

Immunoblotting

For protein isolation, cell pellets were collected, washed in ice cold PBS and lysed in RIPA buffer (150 mM NaCl, 1% Nonidet P-40, 0.5% DOC, 0.1% SDS, 50 mM Tris pH 7.4) with cOmplete Mini Protease Inhibitor Cocktail (Roche). Proteins were quantified using Pierce BCA Protein Assay Kits (Thermo Scientific) and Infinite 200 Pro plate reader (Tecan). Samples were prepared in NuPAGEIDS Sample Buffer (4x;

Invitrogen) containing 50 mM DTT, loaded on NuPAGE 4-12% Bis-Tris Gels (Invitrogen) along with PageRuler Plus Prestained Protein Ladder (Thermo Scientific) and run in NuPAGE MOPS SDS Running Buffer using XCell SureLock Mini-Cell electrophoresis system (Invitrogen). Protein was transferred on iBlot 2 Transfer Stacks (Invitrogen) and blocked in 5% non-fat dried milk in TBS-Tween. For protein detection, nitrocellulose membranes were incubated with primary antibodies overnight at 4°C followed by incubation with secondary HRP-conjugated antibodies for 1 hour at room temperature. SuperSignal West Dura Extended Duration Substrate (Thermo Scientific), Odyssey FC system and ImageStudio (Version 5.5.4, Li-Cor) were used for signal acquisition and protein quantification. Protein of interest levels were quantified relative to housekeeping protein signals. Signal normalization was performed by lane normalization factor or relative to the condition, as indicated. Antibody details are listed in Supplementary Table 2.

mRNA and protein stability

mRNA stability of *UBA1*^{WT} and *UBA1*^{ms} was measured by RT-qPCR using *UBA1*^{WT} and *UBA1*^{ms}-specific primers (Supplementary Table 1), from harvested K562 *SF3B1*^{K700E} cells cultured in complete growth medium with 50 µg/ml actinomycin D (Calbiochem) for the indicated times. NMD of mRNA was assessed by RT-PCR using primers amplifying the *UBA1*^{ms} and *ABCB7*^{MS} junction (Supplementary Table 1), from harvested K562 *SF3B1*^{WT} and *SF3B1*^{K700E} cells cultured in complete growth medium with 100 µg/ml cycloheximide (CHX, Sigma-Aldrich) or DMSO for 4 hours. UBA1 isoform protein stability was assessed by CHX chase in transfected HEK293T cells, treated with 50 µg/ml CHX in complete growth medium. Protein was harvested and UBA1 protein half-life determined by immunoblotting of 2.5 µg or 10 µg total protein with specific antibodies against FLAG-tag, UBA1a/b and Actin (Supplementary Table 2). To stabilize UBA1^{MS} protein, transfected HEK293T cultured with 50 µg/ml CHX were co-treated 10 µg/mL of the proteasome inhibitor MG-132 (MedChemExpress) or DMSO for 6 hours and protein levels were assessed by immunoblotting against FLAG-tag, c-Myc and Actin (Supplementary Table 2).

Polysome Profiling

Cells were pretreated with 100 µg/ml CHX for 5 min prior to lysis to pause translation, washed 2x in ice-cold PBS containing 100 µg/ml CHX and lysed in 800 µl lysis buffer

(20 mM Tris·Cl pH 7.4, 150 mM NaCl, 5 mM $MgCl_2$, 1x protease/phosphatase inhibitor cocktail (Fisher Scientific), 20 units/ml RNAse inhibitor (Thermo Scientific), 100 µg/ml CHX, 1% TX-100, 1 mM DTT, and 24 units/ml TURBO DNase (Thermo Scientific)), directly applied on the dish. Lysates were scraped off the dishes (on ice), incubated on ice for 10 min and homogenized by passing through a 23G needle. Lysates were cleared by centrifugation (13,000 rpm/4ºC/10 min) and equal amounts (circa 12 OD units at 260 nm) were loaded onto 15 to 50% sucrose gradients produced in sucrose gradient buffer (sucrose in 20 mM Tris·Cl pH 7.4, 150 mM NaCl, 5 mM $MgCl_2$, 1x protease/phosphatase inhibitor cocktail, 20 units/ml RNAse inhibitor, and 100 µg/ml CHX) with a gradient forming unit (BioComp). Samples were centrifuged at 36,000 rpm for 3 hours using a SW41Ti rotor in an Optima XE-90 ultracentrifuge (Beckman Coulter). The samples were then analyzed in a piston gradient fractionator (BioComp) using the company's software. 28 sub-fractions were collected at a pace of 2.86 mm per fraction (absorbance at 540 nm) and further used for RNA extraction using a compatible RNA extraction kit (Norgen Biotek). Four and six sub-fractions were combined for analysis of the monosome and polysome fractions, respectively. cDNA synthesis and RT-qPCR were performed as above with 100 ng input and 10 ng Luciferase Control RNA (Promega) spike-in for normalization. Transcript distribution was calculated as the percentage of the target fraction deltaCT values divided by the sum of the deltaCT values.

Viability assays

To assess cell viability, 4×10^4 *SF3B1*^{WT} and *SF3B1*^{K700E} iPSC-HSPCs were seeded in 200 µl StemSpan SFEM II (StemCell Technologies) per well supplemented with 1% P/S, 50 ng/ml of IL3, SCF, FLT3L, and TPO and 10 ng/ml IL6 (PeproTech). Cells were treated with increasing concentrations of TAK-243 or DMSO 0.1% v/v in ultra-low attachment 96-well plates for 24 hours and evaluated for viability by flow cytometry. Alternatively, 5×10^4 *SF3B1*^{WT} and *SF3B1*^{K700E} K562 cells were seeded in 200 µl K562 medium, compound-treated for 72 hours, and evaluated using flow cytometry or luminescence. Cells were stained with 1:200 Aqua and 400 nM Apotracker Green (BioLegend) for 20 min at room temperature, and live cells were defined as Aqua$^-$/Apotracker$^-$ single cells by flow cytometry. For luminescence-based assays, compounds were dispensed onto 384-well plates using a D300e Liquid Dispenser (Tecan). Following incubation (7,500 K562 cells per well in 25 µl medium) and

equilibration (30 min at room temperature), 25 µl of CellTiter-Glo® (Promega) was added, plates were agitated for 2 min (orbital shaker) and luminescence recorded 10 min later using a FLUOstar Microplate Reader (BMG Labtech). Nonlinear 4-Parametric Logistic curves were fitted to the data, and 50% inhibitory concentration (IC_{50}) values were calculated. To assess apoptosis, *SF3B1*^{WT} and *SF3B1*^{K700E} K562 cells were treated with increasing concentrations of TAK-243 for 24 hours followed by immunoblotting for PARP1 and cleaved Caspase-3. Actin was used as loading control. All compounds were dissolved in DMSO (Sigma-Aldrich) at concentrations of 1–10 mM. Cytarabin was purchased from Jena Bioscience, Daunorubicin from Sigma-Aldrich, Doxorubicin from Tocris; and TAK-243, Venetoclax, and Pladienolide B from MedChemExpress.

UBA1 knockdown

Cells were passaged two days prior to knock-down experiments: $2x10^5$ K562 *SF3B1*^{WT} or *SF3B1*^{K700E} cells were transfected with 300 nM Dicer-Substrate Short Interfering RNAs (siRNAs) or negative control using program CM137 on Amaxa 4D-Nucleofector X Unit (Lonza Bioscience).

Competitive growth co-culture

K562 cells were seeded at a total density of $2x10^5$ cells/ml in a 50:50 ratio of *SF3B1*^{WT}:*SF3B1*^{K700E} in 24-well plates, equaling a *SF3B1*^{K700E} VAF of 25%. Cells were treated with 50 nM TAK-243 or DMSO 0.1% v/v for 72 hours, followed by DNA extraction using QIAcube Connect with QIAmp DNA Mini Kit (Qiagen). ddPCR for *SF3B1* VAF was performed as described for primary $CD34^+$ CFU assays. To determine growth curves of individual lines, cells were counted using Guava Muse cell analyzer with Cell Count and Viability Kit (Cytek).

CFU assay of K562 cells

500 cells of K562 *SF3B1*^{WT} or *SF3B1*^{K700E} were seeded per 35 mm dish in Methocult H4434 (StemCell Technologies) containing 15, 32 or 50 nM TAK-243 or DMSO 0.1% v/v. Absolute colony numbers were counted in a blinded fashion after 7 days using an inverted microscope.

Primary CD34⁺ colony-forming unit (CFU) assay with single-colony genotyping

CD34⁺ cells were enriched from BM MNCs of sex-matched *SF3B1*-mutated MDS patients (n = 3) and healthy donors (n = 2) using CD34 Microbeads and positive selection with autoMACS Pro Separator (Miltenyi Biotec). 4000 or 7500 cells were plated in duplicates in MethoCult (H4434; StemCell Technologies) containing 32 nM TAK-243 or DMSO 0.1% v/v and cultured for 14 days. For colony counting, whole culture dishes were imaged using an Eclipse Ti2 inverted microscope (Nikon) in widefield mode and counted using Fiji (PMID: 22743772). For assessment of mutation status, individual colonies were manually and blindly picked under an inverted microscope and DNA isolated using QIAamp DNA Micro Kit (Qiagen). DNA was measured by Nanodrop 2000 Spectrophotometer (Thermo Scientific) and $SF3B1^{K700E}$ variant allele frequency (VAF) by ddPCR (Bio-Rad) in a blinded manner, as previously described.[40] VAF was calculated using QuantaSoft analysis software v.1.7.4 based on Poisson distribution. Control samples containing water, confirmed $SF3B1^{K700E}$ and WT were included in each run. Colonies with *SF3B1* VAF ≥ 40 were scored as mutant; VAF ≤ 4 as WT; and 4 < VAF < 40 as mixed and hence excluded, see Supplementary Table 3.

Supplementary Tables

Supplementary Table 1: List of Primers and Oligos

Target	Sequence	Manufacturer
UBA1 External Control	F: AAGCCGGGTTCTAACTGCTC	Invitrogen
	R: CTTGGCCATTCCGTTGGTTG	
UBA1^{ms} Mis-spliced	F: AAAAACCGGGCCGAGGTATC	Invitrogen
	R: TGCCTGAATGTCCATCCTTCC	
UBA1^{WT} Canonical	F: GTAAAAACCGGGCCGAGGTA	Invitrogen
	R: CCACCACCTGGAAACCACTA	
UBA1 Exon 5-6	F: GTAAAAACCGGGCCGAGGTA	Invitrogen
	R: GATGCCACGGTTGTGACAGA	
ABCB7 Exon 8-9	F: ATGATGCAGGTAATGCTGCT	Invitrogen
	R: TCAGCATAGCCAGAGTAGAGGT	
Firefly luciferase	F: CTCACTGAGACTACATCAGC	Invitrogen
	R: TCCAGATCCACAACCTTCGC	
MYC	F: TCAAGAGGTGCCACGTCTCC	Invitrogen
	R: TCTTGGCAGCAGGATAGTCCTT	
18S Housekeeping	F: GGAGCCTGAGAAACGGCTA	IDT
	R: TCGGGAGTGGGTAATTTGC	
TBP Housekeeping	F: GCACAGGAGCCAAGAGTGAA	Invitrogen
	R: TGTTGGTGGGTGAGCACAAG	
VCL Housekeeping	F: CGATACCACAACTCCCATCAA	Invitrogen
	R: AGCTGCCCTCTCATCAAATAC	
GAPDH Housekeeping	F: AGCCACATCGCTCAGACAC	Invitrogen
	R: GCCCAATACGACCAAATCC	
ACTB Housekeeping	F: GCAAAGACCTGTACGCCAAC	Eurofins
	R: AGTACTTGCGCTCAGGAGGA	
SF3B1 p.K700E c.2098A>G	ddPCR Assay ID dHsaMDS576883070	Bio-Rad
SF3B1 p.R625L c.1874G>T	ddPCR Assay ID dHsaMDS2515478	Bio-Rad
siRNA *UBA1*	DsiRNA *hs.Ri.UBA1.13.2*; 510727692	IDT
siRNA Negative control	DsiRNA Negative control; 510727693	IDT

Supplementary Table 2: List of Antibodies

Antigen	Conjugate	Clone	RRID	Dilution	Manufacturer
CD34	PE-Cy7	4H11	AB_1963576	1:100	Invitrogen
CD45	APC	HI30	AB_10667894	1:100	Invitrogen
CD71	PE	OKT9	AB_10717077	1:100	Invitrogen
CD235a	FITC	HI264	AB_10612923	1:50	BioLegend
UBA1a	N/A	N/A	AB_10984884	1:1000	Invitrogen
UBA1a/b	N/A	N/A	AB_10986095	1:1000	Invitrogen
PARP1	N/A	123	AB_2532215	1:1000	Invitrogen
FLAG	N/A	5A8E5	AB_1720813	1:500	Genscript
Actin	N/A	C4	AB_2335127	1:10000	MPBiomedicals
Lamin B1	N/A	N/A	AB_443298	1:1000	Abcam
cCaspase-3	N/A	5A1E	AB_3076239	1:1000	Cell Signaling
c-MYC	N/A	D84C12	AB_1903938	1:1000	Cell Signaling
GAPDH	HRP	1E2D9	AB_3673986	1:10000	Proteintech
GaR IgG	AF 488	N/A	AB_143165	1:10000	Invitrogen
GaM IgG	HRP	N/A	AB_2536527	1:10000	Invitrogen
GaR IgG	HRP	N/A	AB_2536530	1:10000	Invitrogen
Mouse IgG	N/A	N/A	AB_2929118	N/A	Peprotech
Rabbit IgG	NA	N/A	AB_2722620	N/A	Peprotech

Supplementary Table 3: Patient CFU colony genotyping

Patient	Treatment	CFU ID	VAF	Genotype
**MDS 1**<br>*SF3B1* p.K700E c.2098A>G	DMSO	1	47	mt
		2	47	mt
		3	48	mt
		4	49	mt
		5	53	mt
		6	50	mt
		7	46	mt
		8	39	mixed
		9	49	mt
		10	53	mt
		11	50	mt
		12	52	mt
		13	51	mt
		14	46	mt
		15	48	mt
		16	51	mt
		17	46	mt
		18	3	WT
		19	47	mt
		20	0	WT
		21	45	mt
		22	50	mt
		23	4	mt
		24	49	mt
		25	49	mt
		26	50	mt
		27	40	No amplification
		28	51	mt
		29	58	mt
		30	40	mt
		31	46	mt
		32	50	mt
		33	51	mt
		34	48	mt
		35	47	mt
		36	51	mt
		37	66	No amplification
		38	31	No amplification
		39	50	mt
		40	53	mt
		41	46	mt
		42	45	mt
		43	49	mt
		44	51	mt
		45	52	mt
		46	50	mt
		47	44	mt
		48	51	mt
	TAK-243	1	48	mt
		2	39	mixed
		3	50	mt
		4	51	mt
		5	53	mt
		6	50	mt
		7	53	mt
		8	51	mt
		9	21	mixed
		10	1	WT
		11	38	mixed
		12	58	No amplification
		13	47	mt
		14	51	mt
		15	48	mt
		16	52	mt
		17	63	No amplification
		18	55	mt
		19	49	mt
		20	51	mt
		21	49	mt
		22	21	mixed
		23	1	WT
		24	35	No amplification
		25	39	mixed
		26	2	WT
		27	48	mt
		28	62	mt
		29	0	WT
		30	31	mixed
		31	44	mt
		32	50	mt
		33	44	mt
		34	39	No amplification
		35	33	No amplification
		36	47	mt
		37	46	mt
		38	51	mt
		39	51	mt
		40	53	mt
		41	52	mt
		42	10	mixed
		43	63	No amplification
		44	2	WT
		45	50	mt
		46	47	mt
		47	4	WT

Patient	Treatment	CFU ID	VAF	Genotype
**MDS 2** *SF3B1* p.K700E c.2098A>G	DMSO	1	50	mt
		2	49	mt
		3	49	mt
		4	49	mt
		5	52	mt
		6	51	mt
		7	49	mt
		8	48	mt
		9	50	mt
		10	52	mt
		11	52	mt
		12	49	mt
		13	45	mt
		14	0	WT
		15	48	mt
		16	52	mt
		17	49	mt
		18	48	mt
		19	52	mt
		20	48	mt
		21	0	WT
		22	0	WT
		23	NA	No amplification
		24	51	mt
	TAK-243	1	50	mt
		2	49	mt
		3	0	WT
		4	53	mt
		5	50	mt
		6	58	mt
		7	47	mt
		8	41	mt
		9	49	mt
		10	51	mt
		11	55	mt
		12	0	WT
		13	0	WT
		14	51	mt
		15	54	mt
		16	45	mt
		17	51	mt
		18	53	mt
		19	0	WT
		20	51	mt
		21	47	mt
		22	46	mt
		23	56	mt
**MDS 3** *SF3B1* p.R625L c.1874G>T	DMSO	1	1	WT
		2	47	mt
		3	1	WT
		4	4	mixed
		5	0	WT
		6	47	mt
		7	0	WT
		8	0	WT
		9	3	WT
		10	0	WT
		11	49	mt
		12	1	WT
		13	5	mixed
		14	0	WT
		15	2	WT
		16	33	No amplification
		17	0	WT
		18	49	mt
		19	60	mt
		20	2	WT
	TAK-243	1	0	WT
		2	0	WT
		3	2	WT
		4	9	mixed
		5	10	mixed
		6	11	mixed
		7	37	mixed
		8	3	WT
		9	0	WT
		10	0	WT
		11	0	WT
		12	0	WT
		13	11	mixed
		14	0	WT
		15	0	WT
		16	0	WT
		17	0	WT
		18	0	WT
		19	4	mixed
		20	2	WT
		21	0	WT
		22	1	WT
		23	18	mixed

Supplementary Table 4: Alternative Splice Site Prediction

Bp	Putative splice site	Sequence	Score	Confidence
0	Cryptic donor	TGGTTTCCAGgtatcttggg	10.4	0.7
1	Cryptic acceptor	tggtttccagGTATCTTGGG	7.8	0.4
190	Cryptic acceptor	tttttttgagACGGAGTCTT	6.7	1.0
288	Cryptic acceptor	cctgcctcagCCTCCCGAGT	7.2	0.1
355	Cryptic acceptor	gtatttttagTAGAGACGGG	8.4	0.9
448	Cryptic donor	GGGATTACAGgtgtgagcca	11.4	0.7
450	Cryptic donor	GATTACAGGTgtgagccacc	8.4	0.9
564	Constitutive acceptor	cttgctgcagGCCTCCAGCC	6.9	0.3
635	Cryptic acceptor	ctgtttgtagTCAGTGCGTG	8.6	0.6
847	Cryptic donor	GCTCACCCTGgtggggaagg	10.4	0.9
930	Constitutive donor	TTGGAAGCAGgtgggtgtgg	14.6	0.9
996	Constitutive acceptor	tgtctcacagGGCATGGCAG	7.0	0.6
1070	Constitutive acceptor	ctcctcccagCTACCTCTTC	9.4	0.6
1124	Cryptic acceptor	acccccacagAGTCTGATAC	7.3	0.7
1136	Cryptic donor	CTGATACACAgtaggtgcta	8.3	0.6
1280	Constitutive acceptor	ctctccacagGTGGTGGTGC	11.0	0.9

Supplementary Table 5: Mutational status of MDS patients

ID	Mutation	Variant	VAF (%)	Co-mutations	Sex
1	*SF3B1*	K700E	42	*CUX1, TET2*	M
2	*SF3B1*	K700E	29	*DNMT3A*	F
3	*SF3B1*	R625C	29	None	M
4	*SF3B1*	K700E	43	*DNMT3A*	F
5	*SF3B1*	K666M	42	*TET2, ASXL1, SH2B3, CREBBP*	F
6	*SF3B1*	K666R	35	*KMT2D*	F
7	*SF3B1*	K700E	40	*None*	F
8	*SF3B1*	K700E	38	*DNMT3A, BCORL1, KMT2C*	M
9	*SF3B1*	H662Q	41	*DNMT3A*	M
10	*SF3B1*	K700E	36	*ZNF318, KMT2C, CSNK1A1*	F

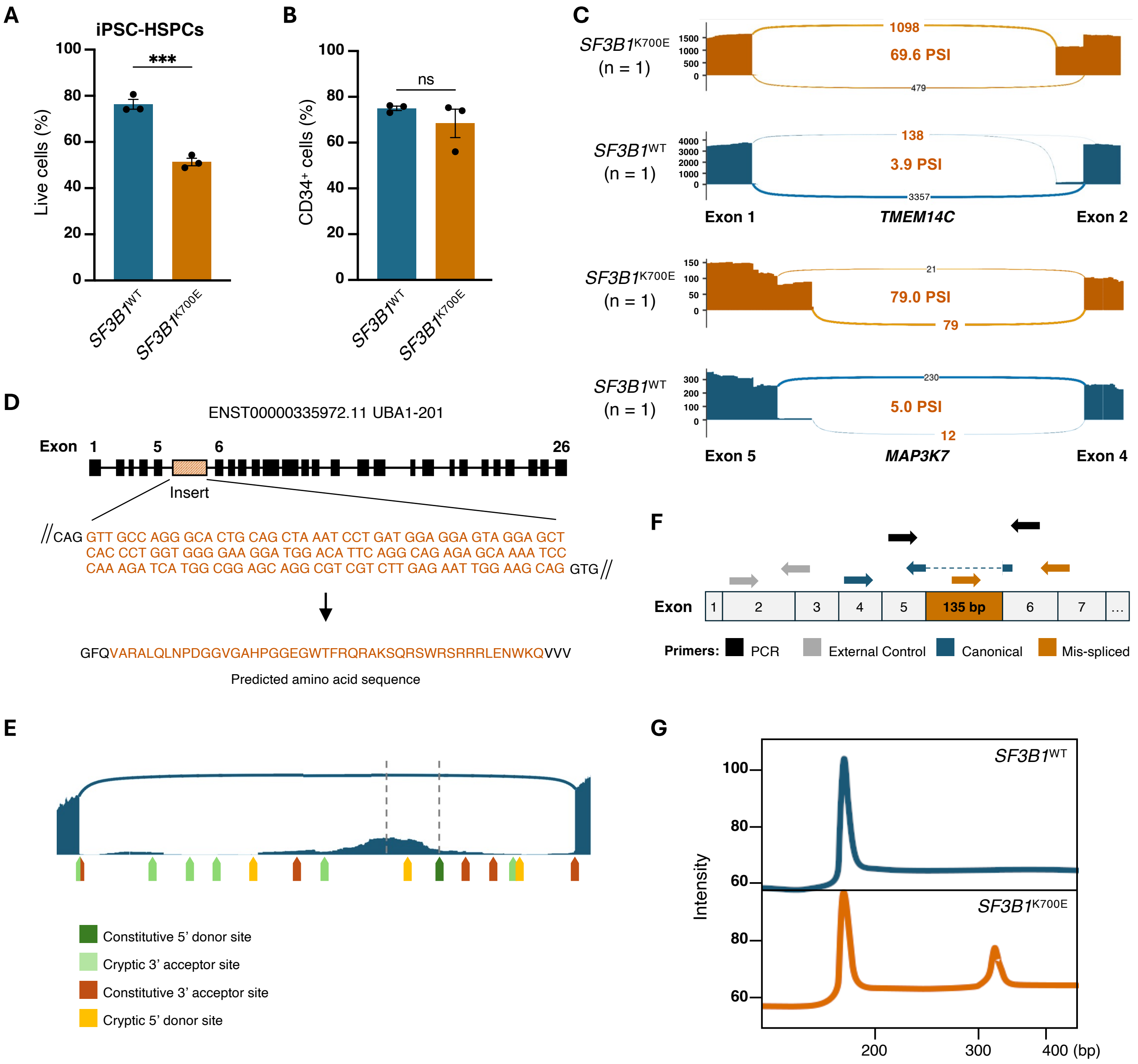

Supplementary Figure 1. (A) Percent viable (n = 3) and (B) CD34⁺ cells (n = 3) from flow cytometry analyses of HSPCs from *SF3B1*^{WT} and *SF3B1*^{K700E} iPSCs after 12 days of differentiation, quantified from main Figure 1B. Mean ± SEM. Unpaired *t*-test. (C) Sashimi plots of known mis-spliced regions in *TMEM14C* and *MAP3K7* in *SF3B1*^{WT} and *SF3B1*^{K700E} from total RNA sequencing of iPSC-derived erythroblasts. Black, canonical splice junction counts; orange, mis-spliced junction counts. (D) Localization of *UBA1* RNA mis-splicing and the predicted nucleotide and amino acid sequences. (E) Sashimi plot of the cryptic exon of *UBA1* depicting 3’ acceptor and 5’ donor sites within intron 5, predicted with cutoff values of 6 and 8 for false acceptor sites and donor sites, respectively. (F) Primer design schematic for the PCR amplification of the *UBA1*^{ms} region (black) and quantification of *UBA1* splice forms by RT-qPCR (gray, blue, orange). (G) Representative lane intensity profile charts from agarose gel electrophoresis of the PCR-amplified exon 5-6 mis-spliced region of *UBA1* from iPSC-derived CD34⁺ cells. Original lane profiles were re-traced and simplified using GIMP image editing software. ***, $P \leq 0.001$; ns, not significant.

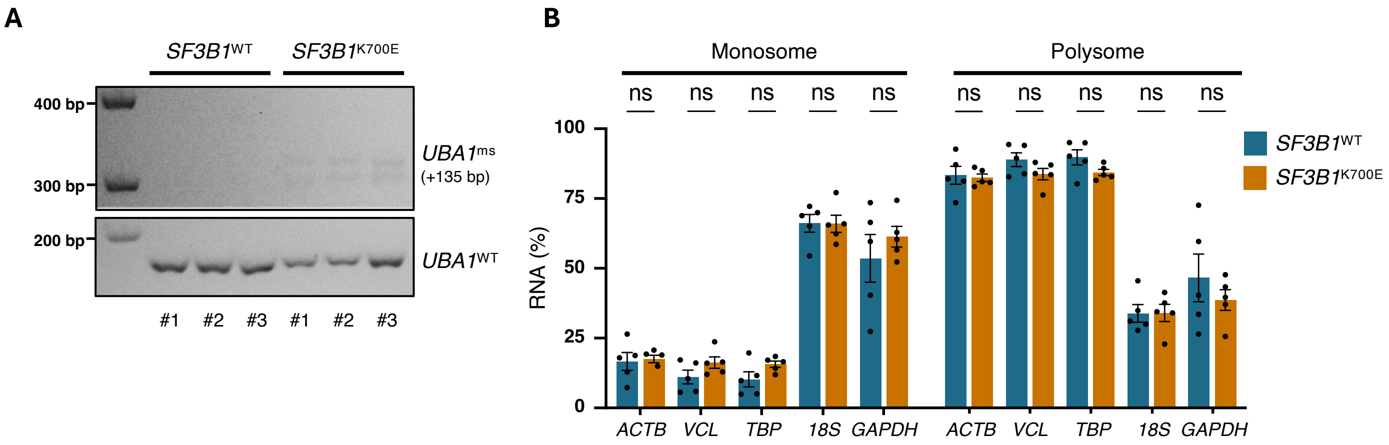

Supplementary Figure 2. (A) Agarose gel electrophoresis of the PCR-amplified exon 5-6 mis-spliced region of *UBA1* from *SF3B1*^{WT} and *SF3B1*^{K700E} K562 cells (n = 3). (B) qPCR analysis of actin *(ACTB)*, vinculin *(VCL)*, TATA-binding protein (*TBP), 18S*, and *GAPDH* transcript distribution in monosome and polysome fractions from *SF3B1*^{WT} and *SF3B1*^{K700E} K562 cells (n = 5). Unpaired *t*-test with Holm-Šídák's multiple comparisons test. ns, not significant.

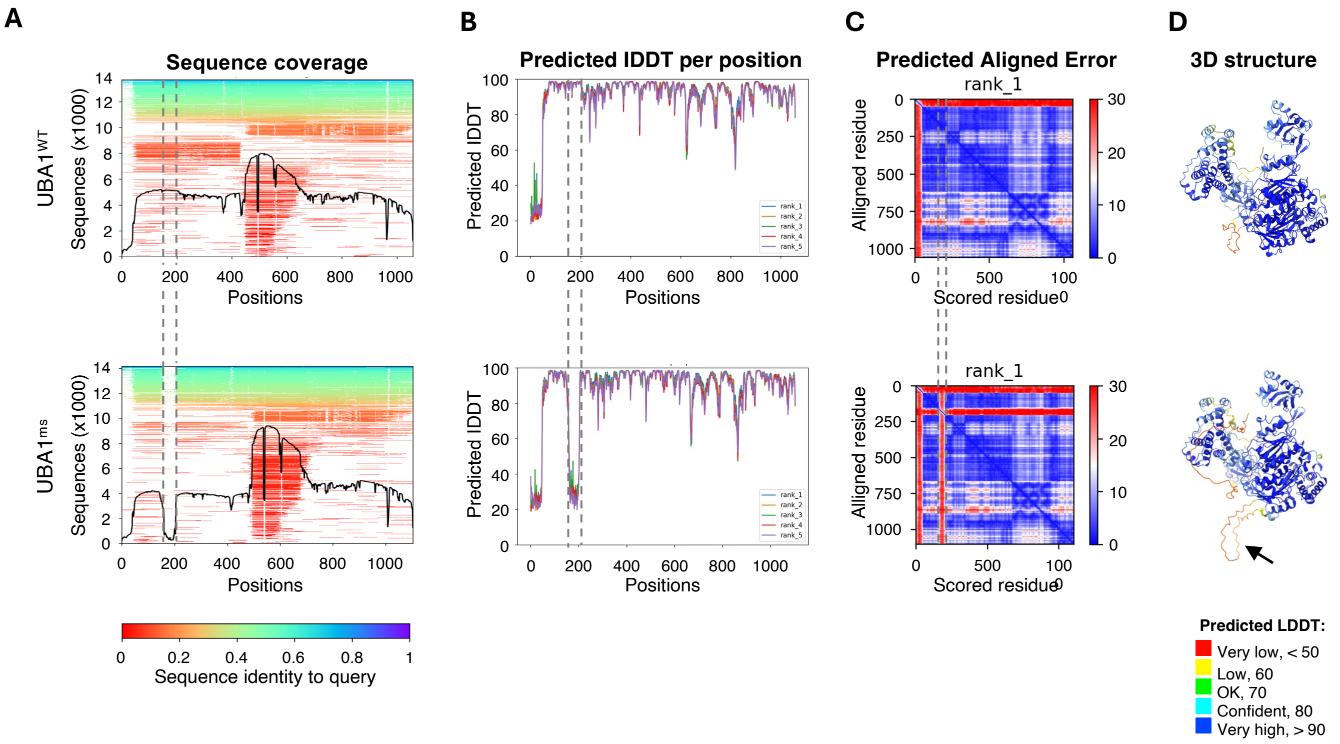

Supplementary Figure 3. (A) Heatmap of multiple-sequence alignment (MSA) with black line qualifying relative coverage from model output of AlphaFold2 structural prediction of UBA1WT and UBA1ms amino acid sequences using ColabFold v1.5.5 (B) Predicted LDDT per residue and (C) Heatmap of predicted alignment plot for the top 5 models obtained. (D) 3D models of predicted secondary structures. Colors reflect local model confidence.

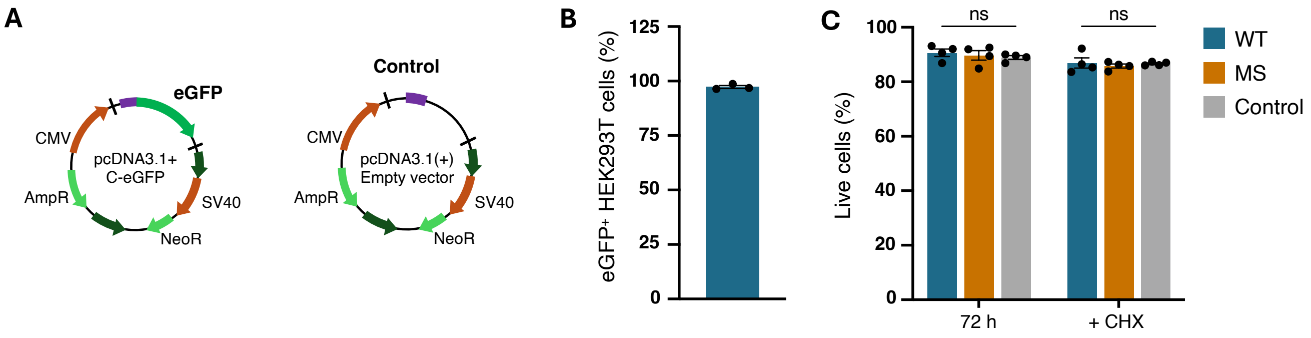

Supplementary Figure 4. (A) Plasmid maps of empty pcDNA3.1 (control) and pcDNA3.1 C-eGFP (eGFP) vectors. (B) Percent eGFP$^+$ HEK293T cells 72 hours post-transfection with a separate eGFP control plasmid, quantified by flow cytometry (n = 3). Mean ± SEM. (C) Percent viable HEK293T cells (n = 4) from flow cytometry analyses 72 hours post-transfection and 12 hours of CHX treatment. Mean ± SEM. Two-way ANOVA with Tukey's multiple comparisons test. ns, not significant.

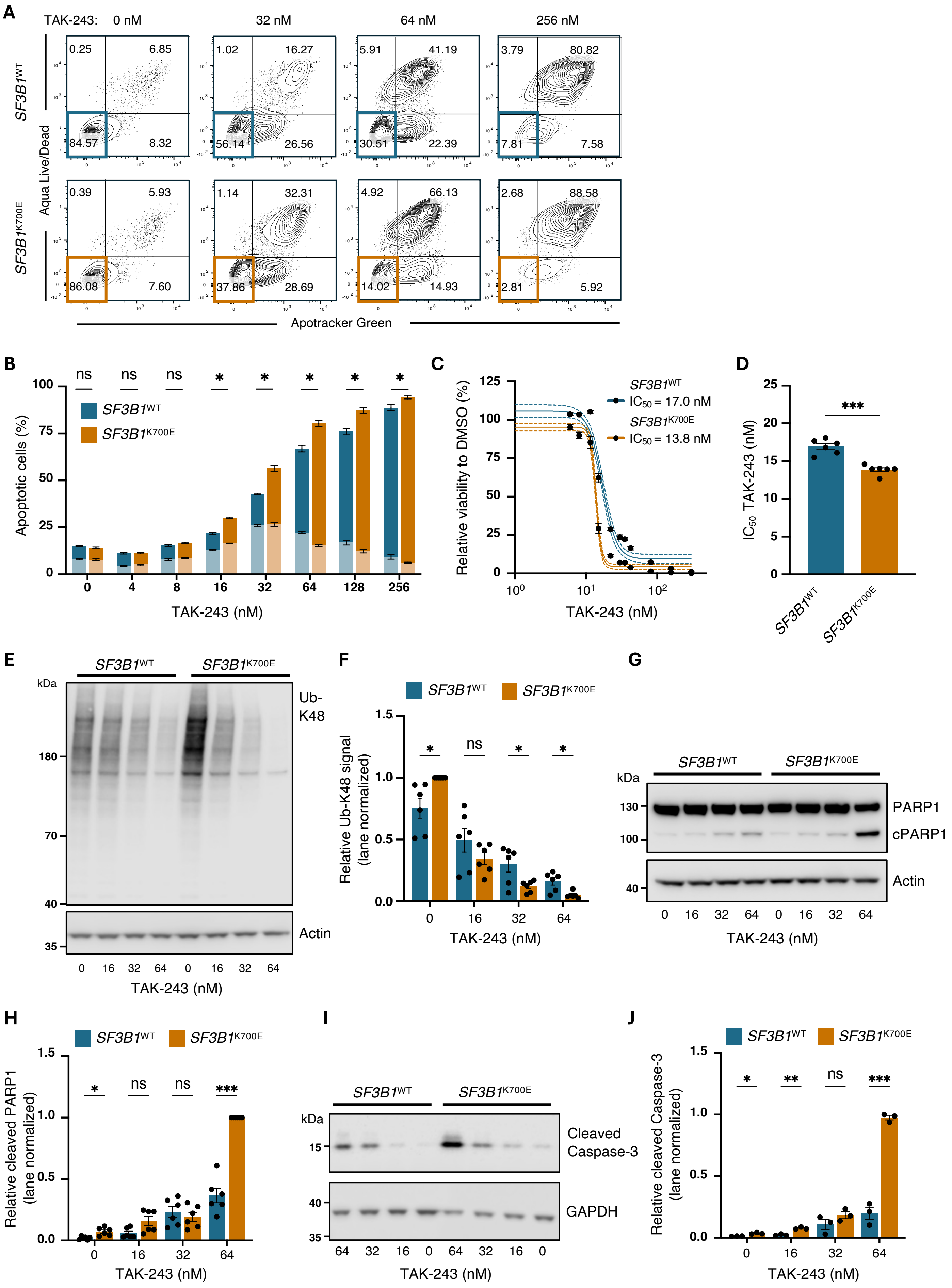

A
TAK-243: 0 nM
32 nM
64 nM
256 nM
SF3B1WT
SF3B1K700E
Aqua Live/Dead
Apotracker Green
0.25
6.85
84.57
8.32
1.02
16.27
56.14
26.56
5.91
41.19
30.51
22.39
3.79
80.82
7.81
7.58
0.39
5.93
86.08
7.60
1.14
32.31
37.86
28.69
4.92
66.13
14.02
14.93
2.68
88.58
2.81
5.92
B
Apoptotic cells (%)
SF3B1WT
SF3B1K700E
ns
ns
ns
*
*
*
*
*
TAK-243 (nM)
C
Relative viability to DMSO (%)
SF3B1WT
IC50 = 17.0 nM
SF3B1K700E
IC50 = 13.8 nM
TAK-243 (nM)
D
IC50 TAK-243 (nM)
***
SF3B1WT
SF3B1K700E
E
SF3B1WT
SF3B1K700E
kDa
Ub-K48
Actin
TAK-243 (nM)
F
SF3B1WT
SF3B1K700E
Relative Ub-K48 signal (lane normalized)
*
ns
*
*
TAK-243 (nM)
G
SF3B1WT
SF3B1K700E
kDa
PARP1
cPARP1
Actin
TAK-243 (nM)
H
SF3B1WT
SF3B1K700E
Relative cleaved PARP1 (lane normalized)
*
ns
ns
***
TAK-243 (nM)
I
SF3B1WT
SF3B1K700E
kDa
Cleaved Caspase-3
GAPDH
TAK-243 (nM)
J
SF3B1WT
SF3B1K700E
Relative cleaved Caspase-3 (lane normalized)
*
**
ns
***
TAK-243 (nM)

Supplementary Figure 5. (A) Representative flow cytometry diagrams of *SF3B1*^{WT} and *SF3B1*^{K700E} K562 cells treated with increasing concentrations of TAK-243 for 72 hours. Colored gates indicate Aqua^{-}/Apotracker^{-} singlets used for quantification of live cells in main Figure 4A-B. (B) Frequency of apoptotic *SF3B1*^{WT} and *SF3B1*^{K700E} K562 cells evaluated by Apotracker and Aqua dead cell stain by flow cytometry after exposure to TAK-243 for 72 hours (n = 3). The fraction of early and late apoptotic cells is displayed in light and dark color, respectively. Early apoptotic cells were defined as Apotracker^{+}/Aqua^{-} and late apoptotic cells as Apotracker^{+}/Aqua^{+} singlets. Mean ± SEM. Unpaired *t*-test. (C) Dose-response curves of *SF3B1*^{WT} and *SF3B1*^{K700E} K562 cells treated with TAK-243 or DMSO for 72 hours (n = 6), assessed by CellTiter-Glo luminescent cell viability assay. Data points represent mean ± SEM signal normalized to control-treated samples. Interpolated sigmoidal, 4PL, standard curves of mean (solid line) and 95% confidence interval (dotted line). IC_{50} of TAK-243 are quantified in (D). Mean ± SEM nM TAK-243. Unpaired *t*-test. (E) Representative immunoblot and (F) quantification of Ub-K48 levels in *SF3B1*^{WT} and *SF3B1*^{K700E} K562 cells treated with increasing concentrations of TAK-243 for 24 hours (n = 6). Lane normalization was used with Actin as a loading control. Mean ± SEM relative signal. Unpaired *t*-test with Holm-Šídák's multiple comparisons test. (G) Representative immunoblot analysis and (H) quantification of cleaved PARP1 (cPARP1) protein levels (lower band) in *SF3B1*^{WT} and *SF3B1*^{K700E} K562 cells treated with TAK-243 for 24 hours (n = 6). Lane normalization was used with Actin as a loading control. Mean ± SEM relative signal. Unpaired *t*-test with Holm-Šídák's multiple comparisons test. (I) Representative immunoblot analysis and (J) quantification of cleaved caspase-3 in *SF3B1*^{WT} and *SF3B1*^{K700E} K562 cells treated with TAK-243 for 24 hours (n = 3). Lane normalization was used with GAPDH as a loading control. Mean ± SEM relative signal. Unpaired *t*-test with Holm-Šídák's multiple comparisons test. *, $P \leq 0.05$; **, $P \leq 0.01$; ***, $P \leq 0.001$; ns, not significant.

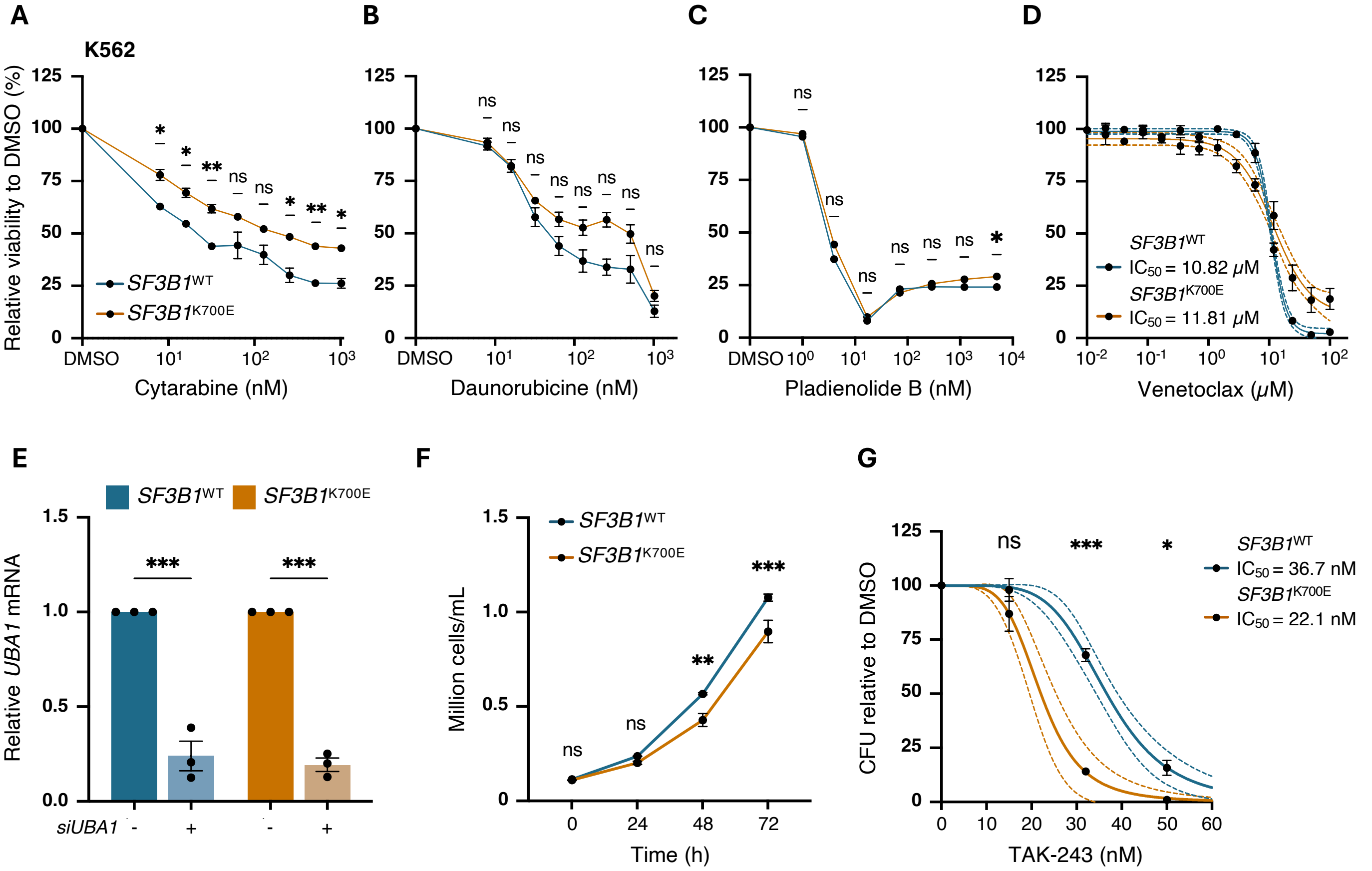

Supplementary Figure 6. Dose-response curves of *SF3B1*^{WT} and *SF3B1*^{K700E} K562 cells treated with increasing concentrations of (A) cytarabine, (B) daunorubicin, (C) pladienolide B, and (D) venetoclax, or DMSO for 72 hours (n = 3, each). Viability was assessed using flow cytometry measurements of Aqua/Apotracker Green stained cells (cytarabine, daunorubicine) or CellTiter-Glo luminescent cell viability assay (pladienolide B, venetoclax). Data points represent mean ± SEM signal normalized to control-treated samples. Interpolated sigmoidal, 4PL, standard curves of mean (solid line) and 95% confidence interval (dotted line, Venetoclax). Unpaired *t*-test with Holm-Šídák's multiple comparisons test. (E) qPCR analysis of total *UBA1* transcript levels in *SF3B1*^{WT} and *SF3B1*^{K700E} K562 cells 48 hours after transfection with *UBA1* siRNA (si*UBA1*) relative to control siRNA-transfected cells (n = 3). Mean ± SEM relative expression. (F) Growth curves for *SF3B1*^{WT} and *SF3B1*^{K700E} K562 cells over 72 hours quantified by automatic cell counting (n = 3). Mean ± SEM million cells/ml. Two-way ANOVA with Šídák's multiple comparisons test. (G) CFU counts of *SF3B1*^{WT} and *SF3B1*^{K700E} K562 cells relative to DMSO-treated conditions, as in Figure 4I. Mean ± SEM relative number of CFUs. Unpaired *t*-test with Holm-Šídák's multiple comparisons test. Interpolated sigmoidal, 4PL, standard curves of mean (solid line) and 95% confidence interval (dotted line). *, $P \leq 0.05$; **, $P \leq 0.01$, ***, $P \leq 0.001$; ns, not significant.

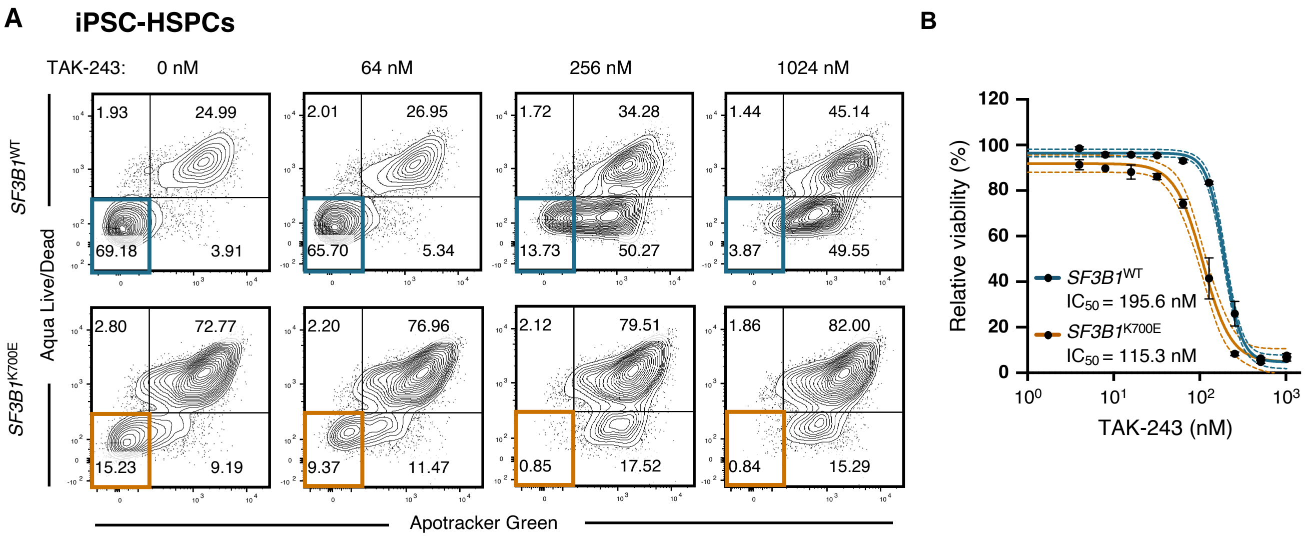

Supplementary Figure 7. (A) Representative flow cytometry diagrams of HSPCs from *SF3B1*^{WT} and *SF3B1*^{K700E} iPSCs treated with increasing concentrations of TAK-243 for 24 hours, showing Aqua$^-$/Apotracker$^-$ live cells used for quantification within the colored gates. (B) Dose-response curves of *SF3B1*^{WT} and *SF3B1*^{K700E} iPSC-derived HSPCs treated with TAK-243 or DMSO for 24 hours. IC_{50} of TAK-243 displayed under the curve are quantified in main Figure 4B (n = 3). Data points represent mean ± SEM signal normalized to control-treated samples. Interpolated sigmoidal, 4PL, standard curves of mean (solid line) and 95% confidence interval (dotted line).

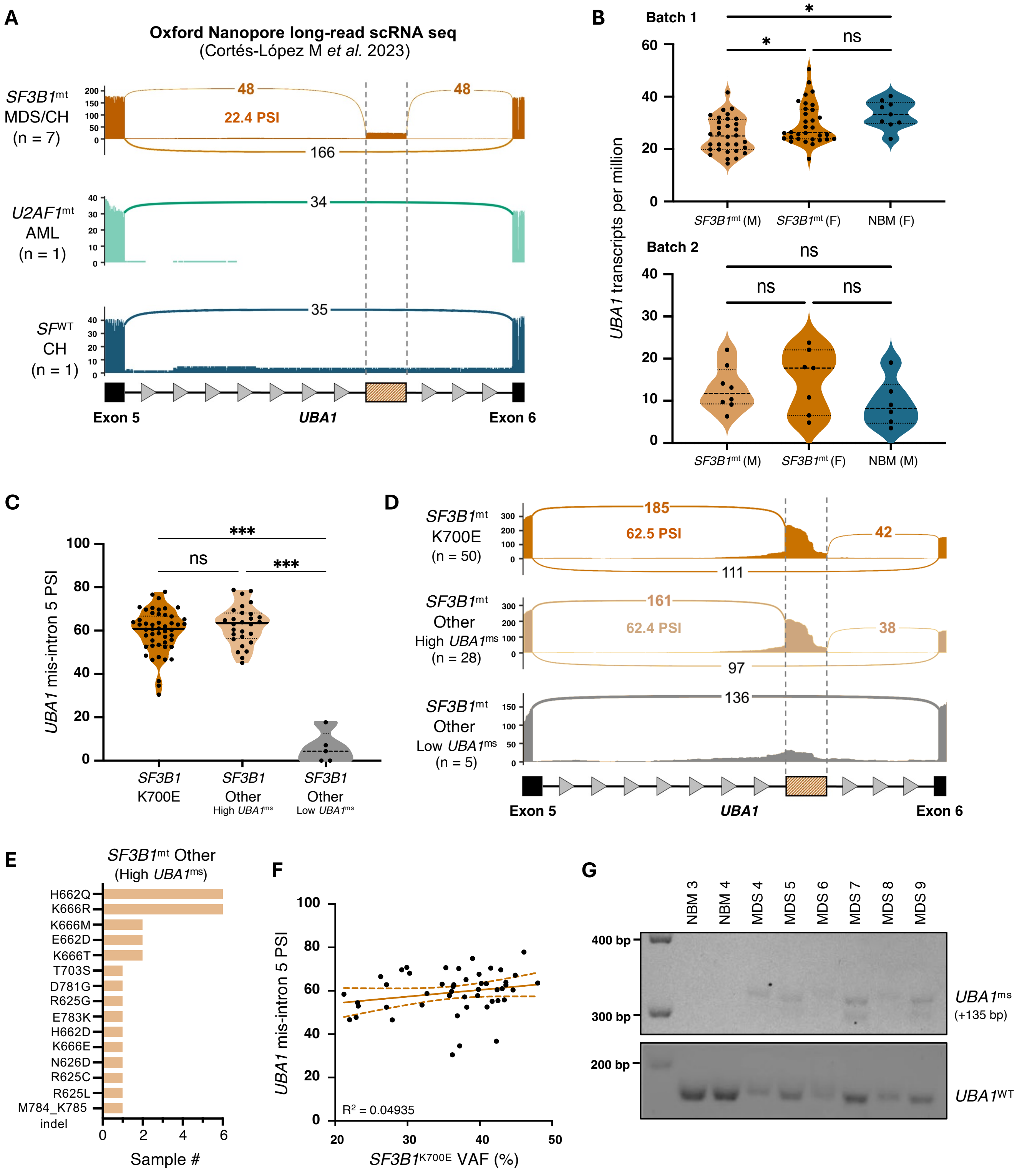
A
Oxford Nanopore long-read scRNA seq
(Cortés-López M et al. 2023)
SF3B1mt MDS/CH (n = 7)
48
22.4 PSI
48
166
U2AF1mt AML (n = 1)
34
SFWT CH (n = 1)
35
Exon 5
UBA1
Exon 6
B
Batch 1
Batch 2
UBA1 transcripts per million
SF3B1mt (M)
SF3B1mt (F)
NBM (F)
NBM (M)
ns
*
C
UBA1 mis-intron 5 PSI
SF3B1 K700E
SF3B1 Other High UBA1ms
SF3B1 Other Low UBA1ms
***
D
SF3B1mt K700E (n = 50)
185
62.5 PSI
42
111
SF3B1mt Other High UBA1ms (n = 28)
161
62.4 PSI
38
97
SF3B1mt Other Low UBA1ms (n = 5)
136
E
SF3B1mt Other (High UBA1ms)
H662Q
K666R
K666M
E662D
K666T
T703S
D781G
R625G
E783K
H662D
K666E
N626D
R625C
R625L
M784_K785 indel
Sample #
F
UBA1 mis-intron 5 PSI
SF3B1K700E VAF (%)
R² = 0.04935
G
NBM 3
NBM 4
MDS 4
MDS 5
MDS 6
MDS 7
MDS 8
MDS 9
400 bp
300 bp
200 bp
UBA1ms (+135 bp)
UBA1WT

Supplementary Figure 8. (A) Sashimi plots for read counts of the *UBA1*^{ms} region from published RNA sequencing data of *SF3B1*^{mt} MDS/CH (n = 7), *U2AF1*^{mt} AML (n = 1) and *SF*^{WT} CH (n = 1) cases.[46] (B) Violin plots of *UBA1* transcript per million counts in *SF3B1*-mutated and NBM samples from the sequenced cohort separated by sequencing batch (Batch 1: *SF3B1*^{mt} (M), n = 32, *SF3B1*^{mt} (F), n = 32, NBM (F), n= 9; Batch 2: *SF3B1*^{mt} (M), n = 8, *SF3B1*^{mt} (F), n = 7, NBM (F), n= 6). One-way ANOVA with Tukey's multiple comparisons test. (C) Violin plots of PSI *UBA1* intron 5 mis-splicing in *SF3B1*-mutated cases, comparing *SF3B1*^{K700E} to all other *SF3B1* variants with high (≥20% PSI) or low (≤20% PSI) *UBA1*^{ms} (n^{K700E} = 50; n^{High} = 28; n^{Low} = 5). One-way ANOVA with Tukey's multiple comparisons test. (D) Sashimi plots for read counts of the *UBA1*^{ms} region in $CD34^+$ BM MNCs of MDS-*SF3B1* patients, grouped by *SF3B1*^{K700E} or other variants with either high or low *UBA1* mis-intron 5 PSI. (E) *SF3B1* variants other than K700E of cases with high *UBA1*^{ms}. (F) Scatter plot of *UBA1* intron 5 mis-splicing PSI over *SF3B1*^{K700E} VAF in *SF3B1*^{K700E} cases (n = 48). Simple linear regression (solid line) with 95 CI (dotted line). (G) Agarose gel electrophoresis of the PCR-amplified exon 5-6 mis-spliced region of *UBA1* from patient (MDS, n = 6) or healthy donor-derived (NBM, n = 2) BM MNC $CD34^+$ cells. Lower band corresponds to the PCR product of canonically spliced, and upper band to mis-spliced *UBA1*. *, $P \leq 0.05$; ***, $P \leq 0.001$; ns, not significant. CH, clonal hematopoiesis; AML, acute myeloid leukemia.
